# Supplementary material for: Spatial gene regulatory networks driving cell state transitions during human liver disease
Source: EMBO Mol Med. 2025 Apr 25;17(6):1452–74. doi: 10.1038/s44321-025-00230-6 (PMC12162837; doi:10.1038/s44321-025-00230-6)
Supplement: Supplementary file 1 — Table EV1 [file 44321_2025_230_MOESM1_ESM.pdf]

Visium sample ID	Manuscript nomenclature	Patient ID	Diagnosis	Age	Ethnicity	Sex (M/F)	Surgical details	Background Information
**V10M16-070-A1**	Sample a	MC2	HCC	71	White-British	M	hepatectomy for large HCC	Alcohol-induced cirrhosis / child-pugh A6 at time of operation
**V10M16-070-B1**	Sample b	MC5/12	HCC	68	White-British	M	resection for HCC	Alcohol-induced cirrhosis / child-pugh A5 at time of operation
**V10M16-070-C1**	Sample c' (technical replicate)	MC3/09	HCC	58	White-British	M	resection for HCC	Left lobe hepatoma / alcohol-induced cirrhosis / child-pugh A6 at time of operation
**V10M16-070-D1**	Sample c							

Visium Sample ID	Manuscript nomenclature	Patient ID	Diagnosis	Age (years)	Ethnicity	Sex (M/F)	Ethanol (ml)	BMI	Fibroscan (Kpa)	Steatosis grade (CAP)	Fibrosis stage (Ishak)	FIB-4	Co-morbidities
**V11D13-083_A1**	Sample d	sample d	MASLD	69	East Asian	M	<20	30.45	9.5	S3	IS3	N/D	alpha-thalassaemia / hypertension
**V11D13-083_B1**	Sample e	sample e	MASLD	52	White-British	M	0	46.2	30.8	S2	IS4	2.01	T2DM / hypertension / asthma
**V11D13-083_C1**	Sample f	sample f	MASLD	73	White-British	M	70	28.2	17.3	S1	IS4 - 5	2.66	T2DM / hypertension / ischaemic heart disease
**V11D13-083_D1**	Sample g	sample g	MASLD	63	White-British	M	100	35	18.4	S2-S3	IS6	1.23	T2DM/ hypertension / rheumatoid arthritis
		IMC	MASLD	59	White-British	M	80	32.6	13.1	S1	IS3-4	0.68	T2DM/ hypertension

	Manuscript nomenclature
**GSM5764426**	H36
**GSM5764428**	H38

Table EV1. Clinical sample information.
