# Supplementary material for: Spatial gene regulatory networks driving cell state transitions during human liver disease
Source: EMBO Mol Med. 2025 Apr 25;17(6):1452–74. doi: 10.1038/s44321-025-00230-6 (PMC12162837; doi:10.1038/s44321-025-00230-6)
Supplement: Supplementary file 2 — Appendix [file 44321_2025_230_MOESM2_ESM.pdf]

Appendix

Spatial gene regulatory networks driving cell state transitions during human liver disease

Nigel L Hammond[1], Syed Murtuza Baker[1], Sokratia Georgaka[1], Ali Al-Anbaki[1], Elliot Jokl[1], Kara Simpson[1], Rosa Sanchez-Alvarez[1], Varinder S Athwal[1,2], Huw Purssell[1,2], Ajith K Siriwardena[1,2], Harry VM Spiers[3], Mike J Dixon[1], Leoma D Bere[1], Adam P Jones[1], Michael J Haley[1], Kevin N Couper[1], Nicoletta Bobola[1], Andrew D Sharrocks[1], Neil A Hanley[1,4], Magnus Rattray[1] & Karen Piper Hanley[1]

This PDF file includes:

- Appendix Figures S1 to S24
- Appendix Table S1

Table of Contents

Appendix Figure S1. ST resolves the patho-architecture of human liver tissue from patients diagnosed with cirrhosis. 3

Appendix Figure S2. Visium Tissue Optimisation Assay. 4

Appendix Figure S3. Visium Spatial transcriptomics data (experimental cohort) for human liver tissue. 5

Appendix Figure S4. Spatial mapping of scar associated gene ontology terms 6

Appendix Figure S5. snRNA-seq and snATAC-seq count metrics 7

Appendix Figure S6. Integrative analysis of enhancer-promoter interactions with peak2gene linkage. 8

Appendix Figure S7. snRNA-seq integration of healthy and cirrhotic human liver. 9

Appendix Figure S8. Visium Spatial transcriptomics data (validation cohort) for human fibrotic liver. 10

Appendix Figure S9. Endothelial cell subpopulations in healthy and cirrhotic liver. 11

Appendix Figure S10. Quantification of spatial expression signatures in healthy and disease liver. ... 12

Appendix Figure S11. Mesenchymal cell subpopulations in healthy and cirrhotic liver. 13

Appendix Figure S12. Macrophage cell subpopulations in healthy and cirrhotic liver. 14

Appendix Figure S13. Lymphocyte cell subpopulations in healthy and cirrhotic liver. 15

Appendix Figure S14. Parenchymal cell subpopulations in healthy and cirrhotic liver. 16

Appendix Figure S15. Deconvolution and spatial mapping of cell type sub-clusters in sample A. 17

Appendix Figure S16. Co-location analysis of cell type sub-clusters in sample A. 18

Appendix Figure S17. Deconvolution and spatial mapping of cell type sub-clusters in sample C. 19

Appendix Figure S18. Co-location analysis of cell type sub-clusters in sample C. 20

Appendix Figure S19. Scar and scar-interface associated co-location factors across experimental cohort patient samples. 21

Appendix Figure S20. Non-parenchymal cell sub-populations are co-localised within the fibrotic niche. 22

Appendix Figure S21. Disease-associated hybrid parenchymal cells are co-localised to the scar-interface. 23

Appendix Figure S22. SOX9 and TROP2 localisation in human development and adult fibrotic liver. .. 24

Appendix Figure S23. Characterisation of spatial features with imaging mass cytometry (IMC). 25

Appendix Figure S24. Gene expression driving disease associated hepatocytes. 26

Appendix Table S1. 27

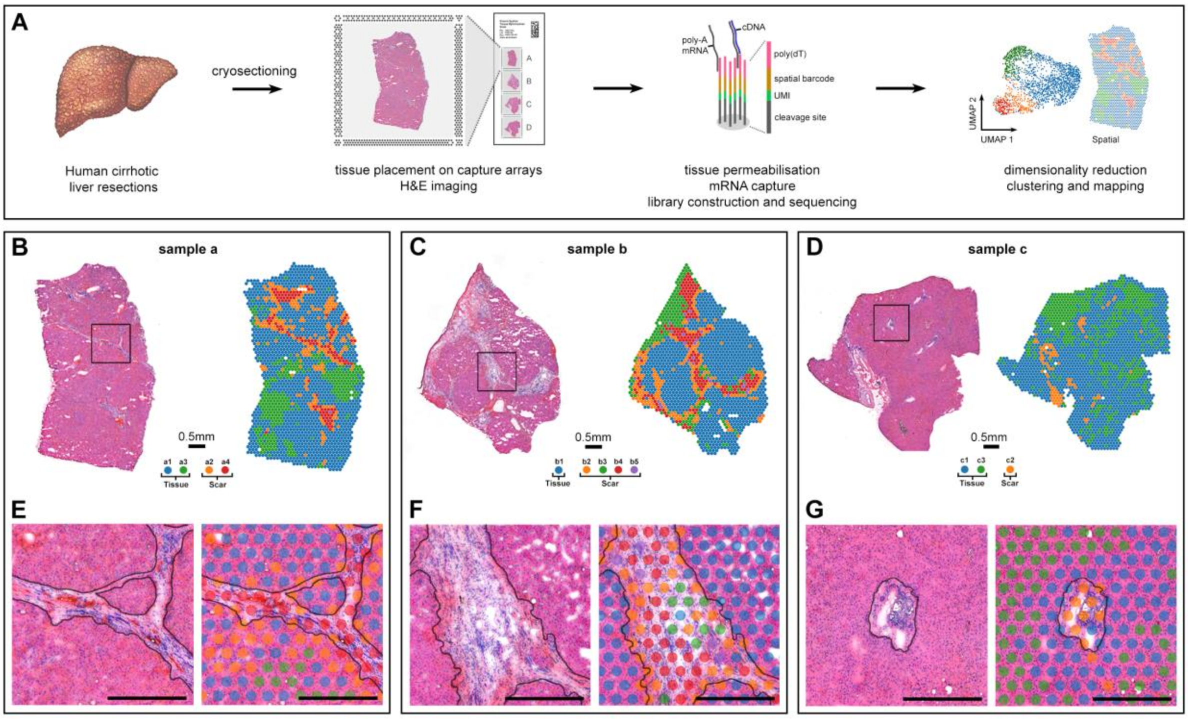

Appendix Figure S1. ST resolves the patho-architecture of human liver tissue from patients diagnosed with cirrhosis.

(**A**) Schematic of the Visium spatial transcriptomics (ST) workflow. (**B-D**) H&E stained cryosections and the associated spatial cluster assignments (*k*-means) for three patients diagnosed with liver cirrhosis. (**E-G**) High resolution projections of clustered spots from indicated regions (box) define liver parenchyma (blue) from fibrotic scars (red) and the interface between them (orange). Spatial spots (55 µm) represent gene expression profiles underlying the tissue, colour denotes cluster. Scale bar, 500 µm.

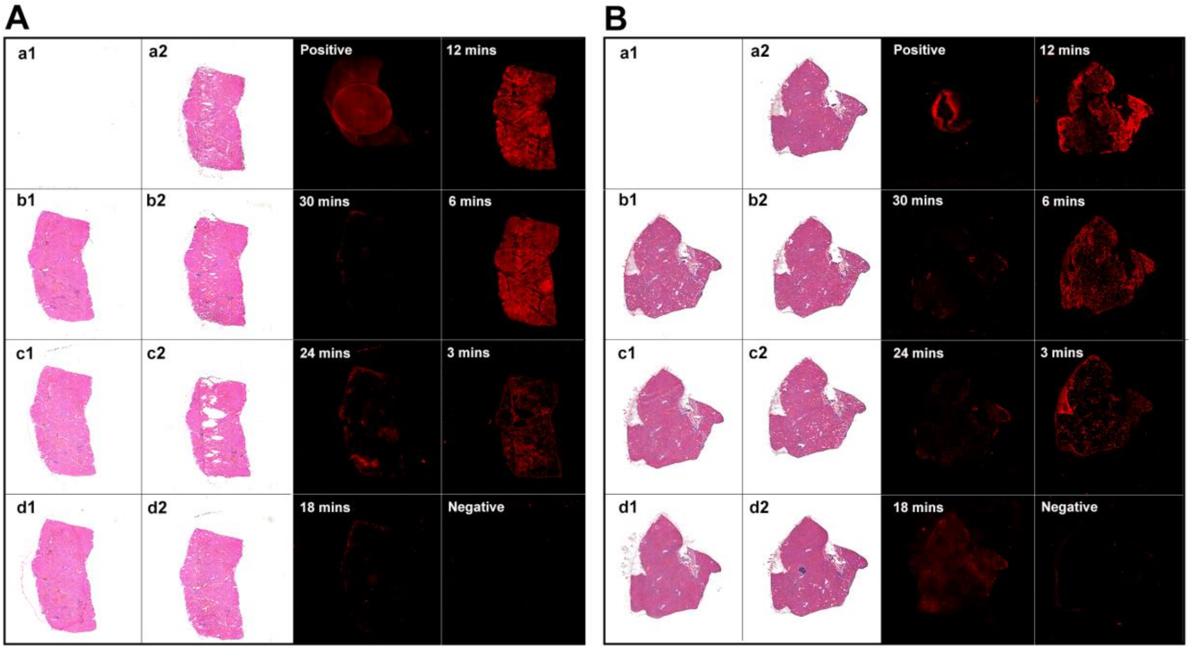

Appendix Figure S2. Visium Tissue Optimisation Assay.

(**A**, **B**) Serial cryosections from two independent human liver samples were placed on capture areas of Visium Optimisation slides (**A**; a1-d2, **B**; a1-d2) and imaged following histological staining. A permeabilization timeseries (0-30 mins) followed by fluorescent cDNA synthesis and imaging, shows the peak fluorescent signal is achieved after 12 minutes. Positive control, mouse cDNA; negative control, minus permeabilization enzyme.

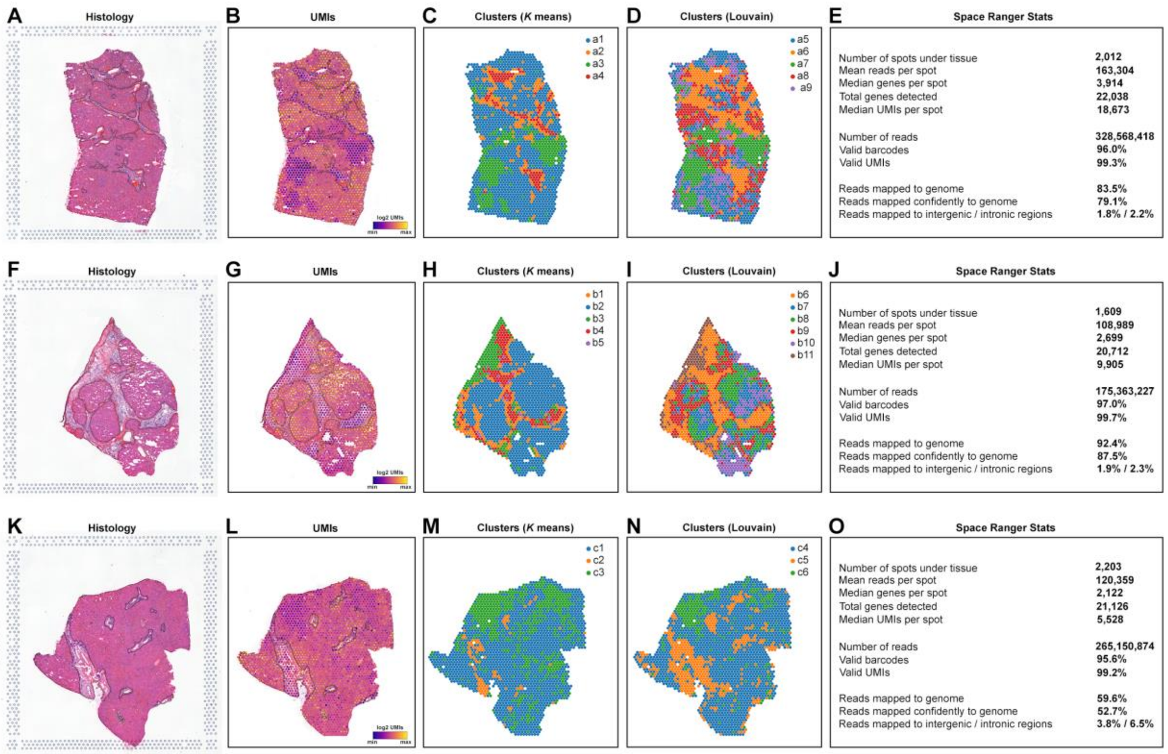

Appendix Figure S3. Visium Spatial transcriptomics data (experimental cohort) for human liver tissue.

(**A**-**O**) Summary of spatial mapping data for human patient samples diagnosed with liver cirrhosis; *sample a* (**A**-**E**), *sample b* (**F**-**J**) and *sample c* (**K**-**O**). Brightfield imaging (**A**, **F**, **K**), spatial mapping of unique molecular identifiers (UMIs) (**B**, **G**, **H**), spatial clusters (**C**, **D**, **H**, **I**, **M**, **N**) and Space Ranger mapping statistics (**E**, **J**, **O**) for each sample.

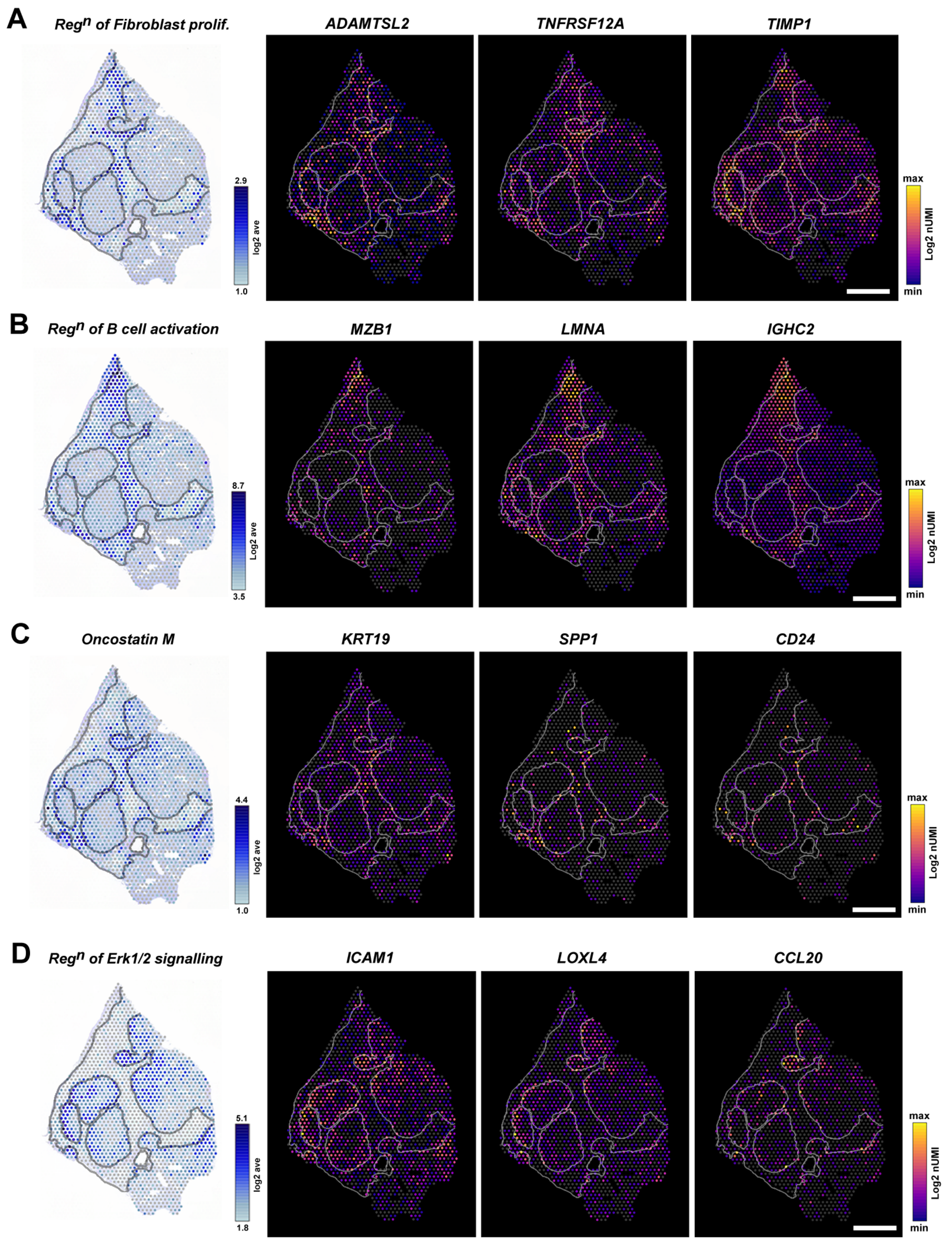

Appendix Figure S4. Spatial mapping of scar associated gene ontology terms .

(**A-D**) Spatial expression of gene modules (log2 average UMIs) underlying GO terms with corresponding spatial expression of select genes (log2 UMIs). Scale bars, 1mm.

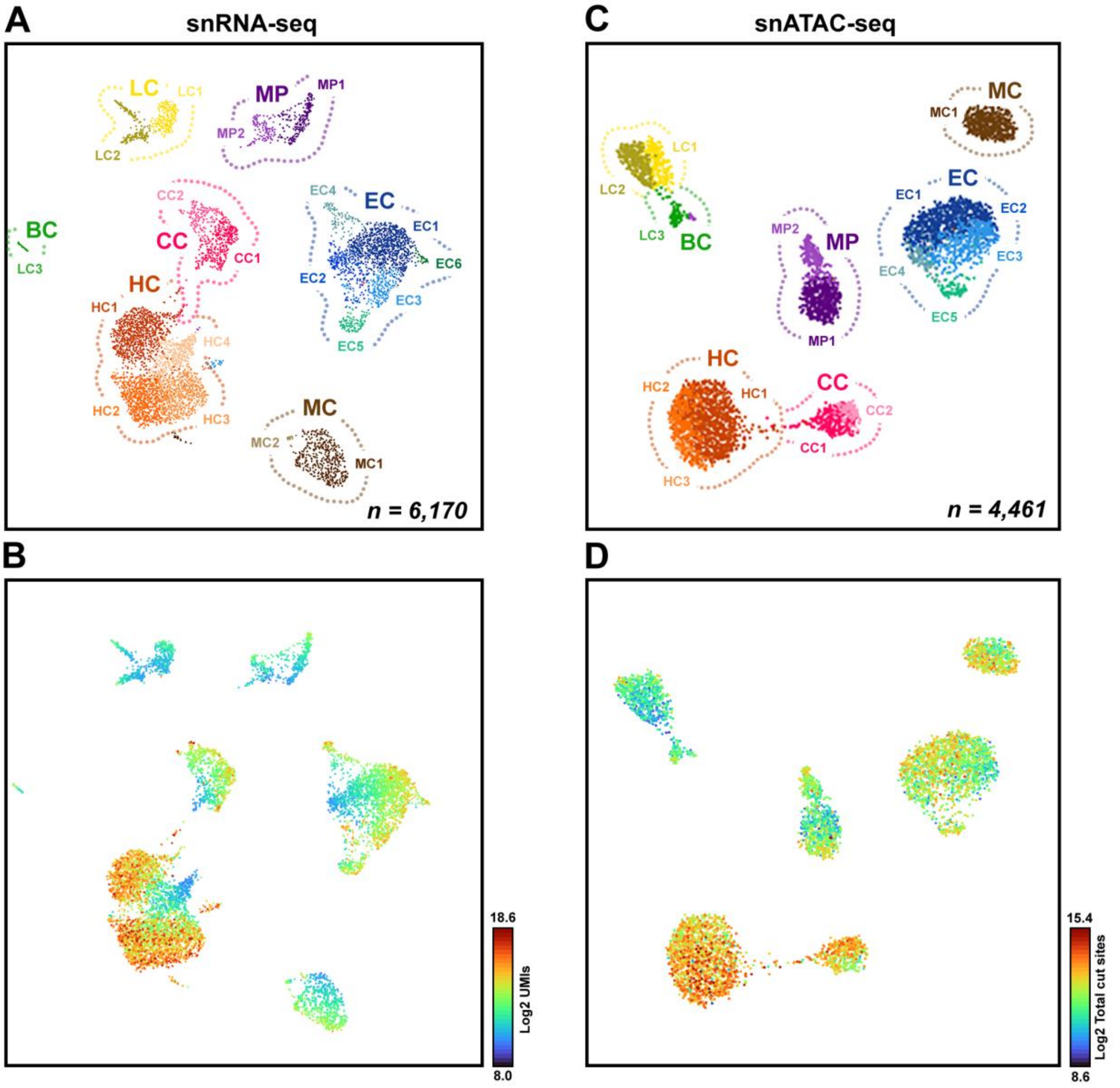

Appendix Figure S5. snRNA-seq and snATAC-seq count metrics

(**A**,**B**) UMAP plots of cirrhotic snRNA-seq sub-clusters and associated unique molecular identifier counts per cell (log2 UMIs). (**C**,**D**) UMAP plots of cirrhotic snATAC-seq sub-clusters and associated total cut sites per cell (log2 total cuts). Each spot represents individual nuclei, *n* denotes number of nuclei profiled. Hepatocytes (HC), endothelial cells (EC), cholangiocytes (CC), macrophages (MP), mesenchymal cells (MC), NK/T Lymphocytes (LC), B lymphocytes (BC).

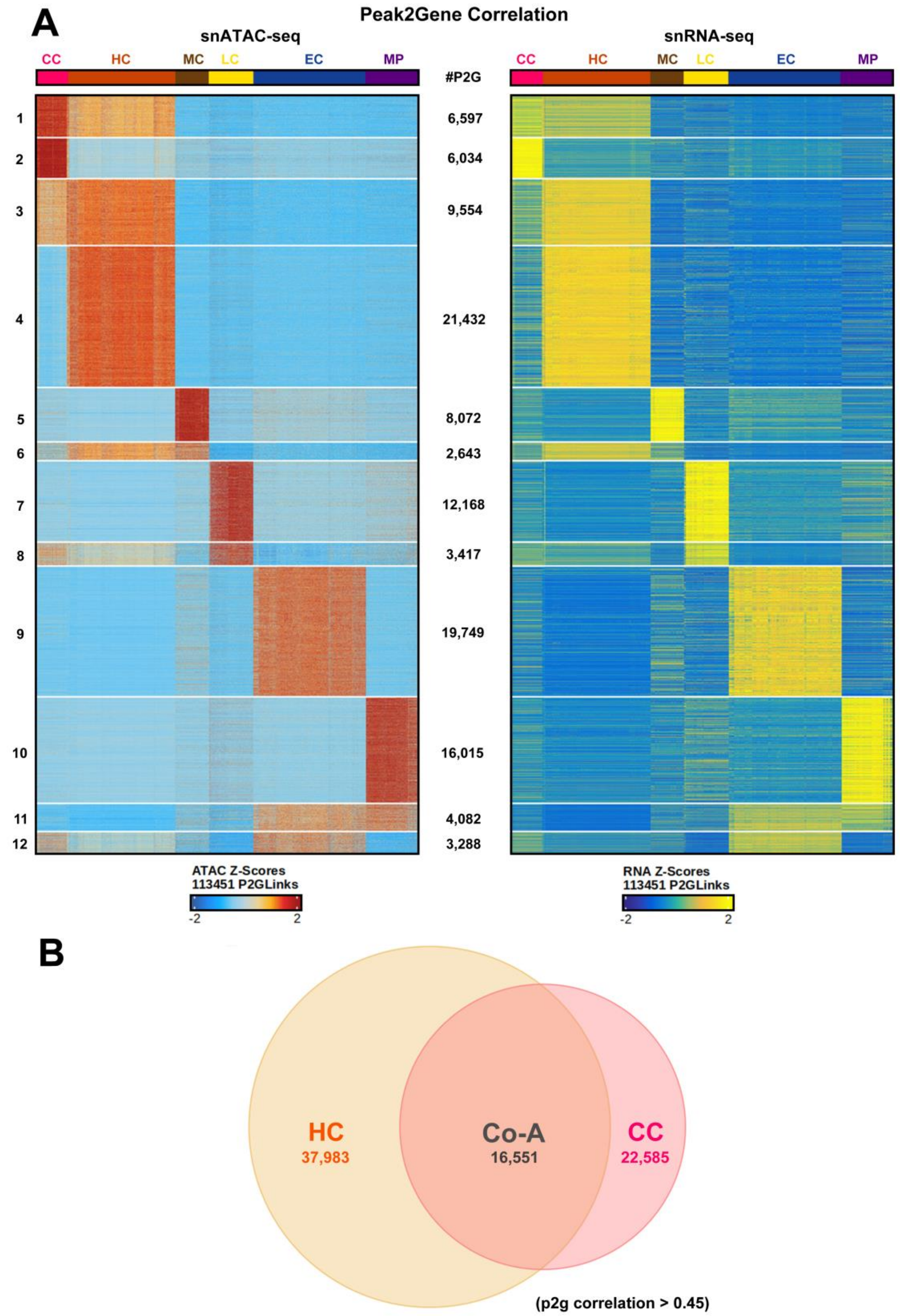

Appendix Figure S6. Integrative analysis of enhancer-promoter interactions with peak2gene linkage.

(**A**) Peak2gene heatmaps show correlation of 113,451 co-accessible peaks (snATAC-seq) linked to promoter gene expression (snRNA-seq) with a genomewide cutoff of 0.45 and rows clustered by *k*-means (12). (**B**) Venn diagram shows the proportion of unique and shared co-accessible peak2gene links (clusters 1-4) between hepatocytes (HC) and cholangiocytes (CC).

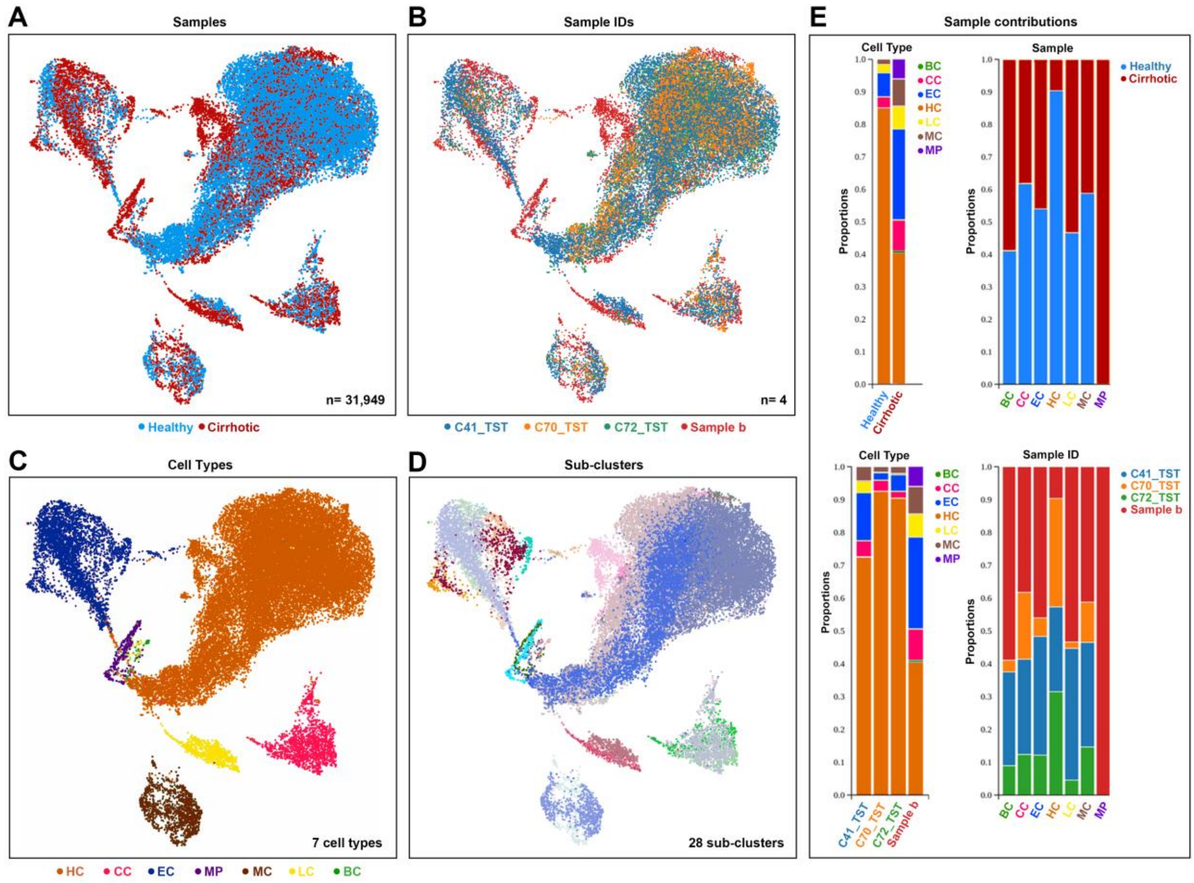

Appendix Figure S7. snRNA-seq integration of healthy and cirrhotic human liver.

(**A-D**) UMAP plots with metadata showing (**A**) healthy (blue) and cirrhotic (red) samples, (**B**) patient sample IDs, (**C**) broad cell types, and (**D**) all cell sub-clusters. (**E**) Stacked bar plots to show cell type and sample contributions (%) for integrated snRNA-seq. Each spot represents individual nuclei, *n* denotes number of nuclei profiled. Hepatocytes (HC), endothelial cells (EC), cholangiocytes (CC), macrophages (MP), mesenchymal cells (MC), NK/T Lymphocytes (LC), B lymphocytes (BC)

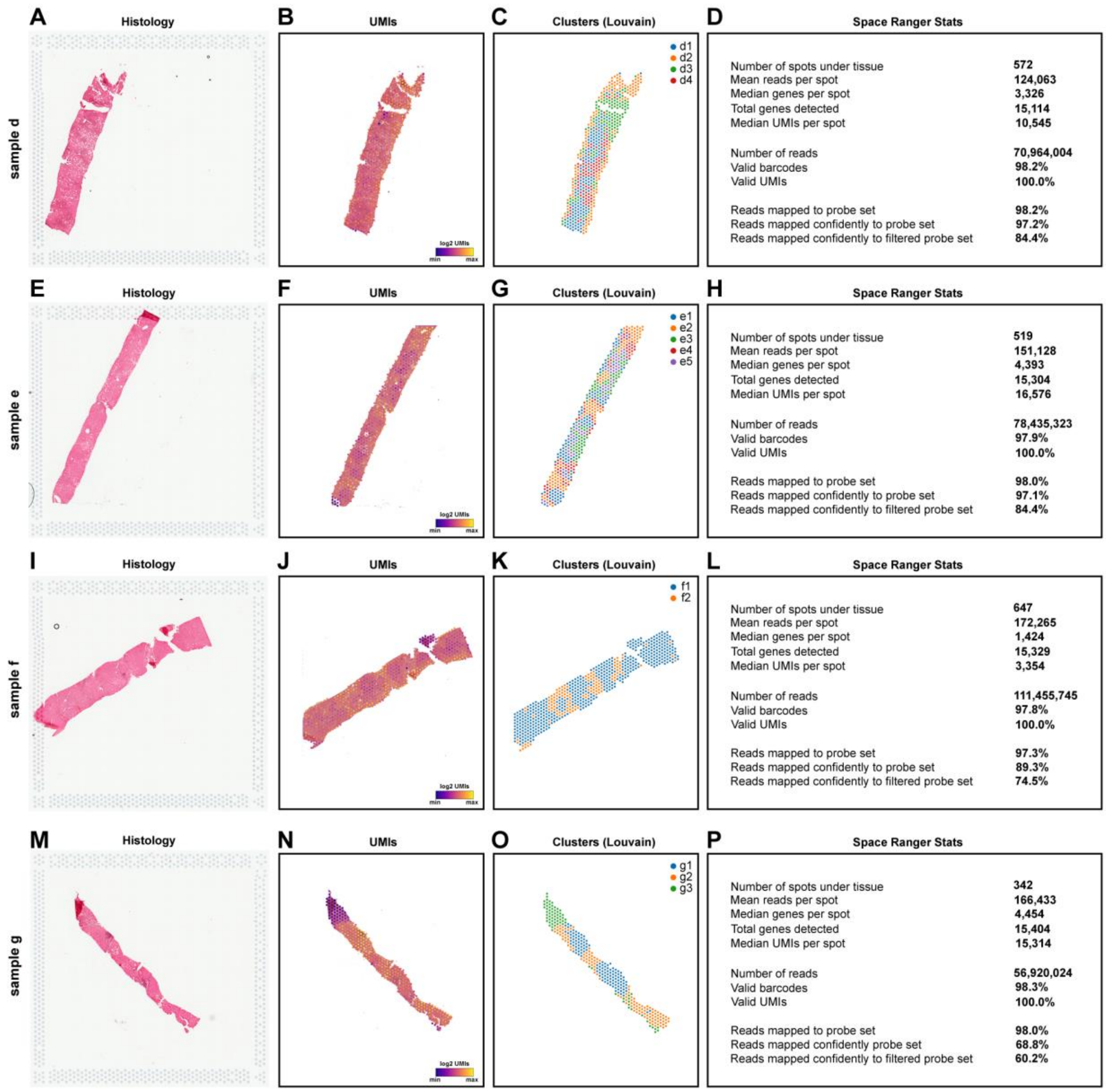

Appendix Figure S8. Visium Spatial transcriptomics data (validation cohort) for human fibrotic liver. (**A**-**P**) Summary of spatial mapping data for fibrotic liver *sample d* (**A**-**D**), *sample e* (**E**-**H**), *sample f* (**I**-**L**) and *sample g* (**M-P**). Brightfield imaging (**A**, **E**, **I**, **M**), spatial mapping of unique molecular identifiers (UMIs) (**B**, **F**, **J**, **N**), spatial clusters (**C**, **G**, **K**, **O**) and Space Ranger mapping statistics (**D**, **H**, **L**, **P**) for each sample. Each spatial spot is 55 µm.

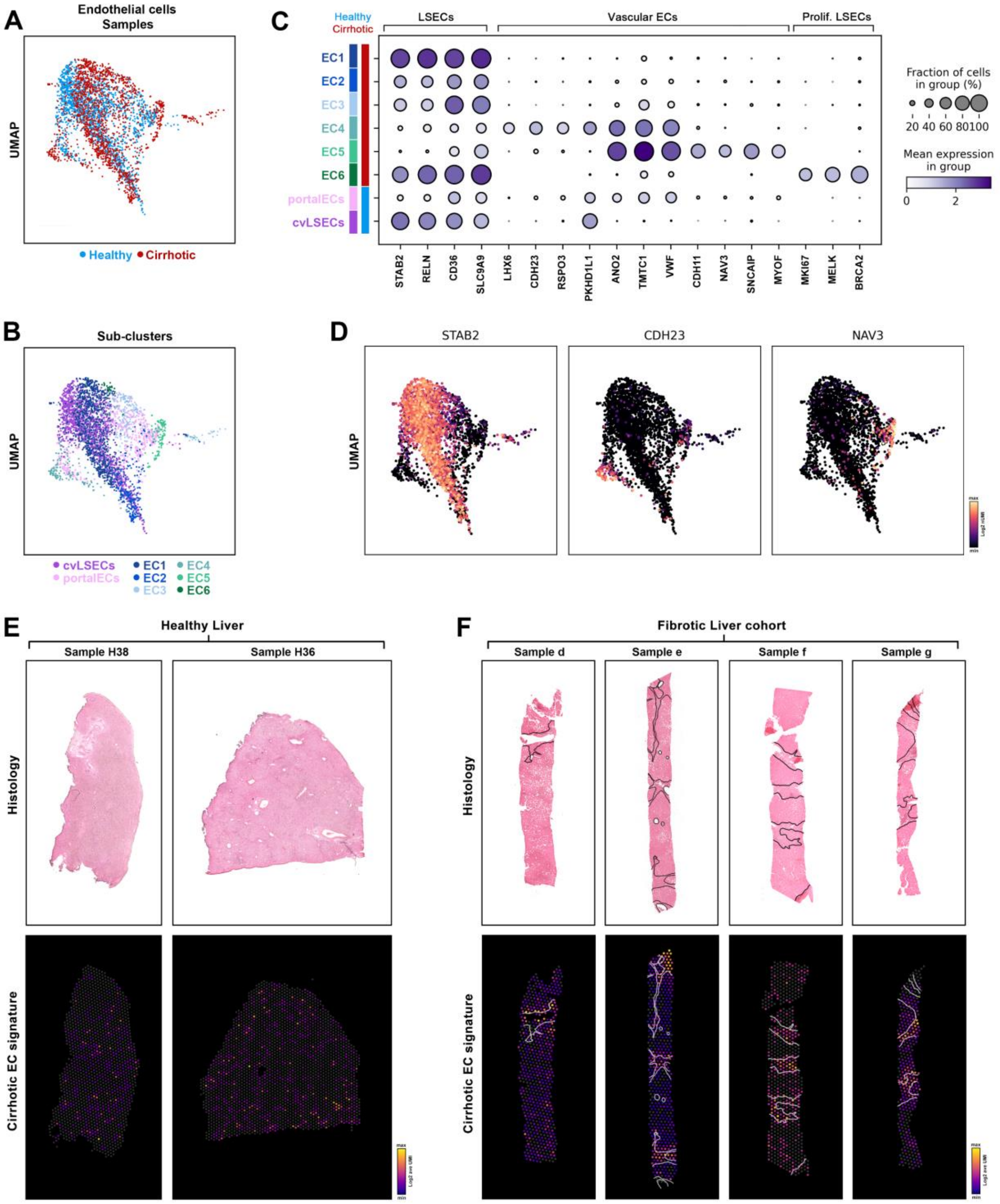

Appendix Figure S9. Endothelial cell subpopulations in healthy and cirrhotic liver.

(**A**,**B**) UMAP plots of (**A**) endothelial cells (ECs) across healthy and cirrhotic samples and (**B**) cells coloured by sub-cluster metadata. (**C**) Dot plot showing mean expression of genes associated with EC sub-clusters and how they compare to healthy EC sub-populations. (**D**) Expression UMAPs of *STAB2*, *CDH23* and *NAV3 (*log2 UMIs*)*. Spatial mapping of cirrhotic EC signature (average UMIs of *LHX6*, *CDH23*, *RSPO3*, *PKHD1L1*, *CARMIL1*, *ANO2*, *VWF*, *TMTC1*, *SNCAIP*, *CDH11*, *NAV3*, *MYOF*) in (**E**) healthy and (**F**) fibrotic validation samples. UMAP plots represent individual nuclei (**A**,**B**,**D**), spatial spots (55 µm) represent area of tissue profiled (**E**,**F**).

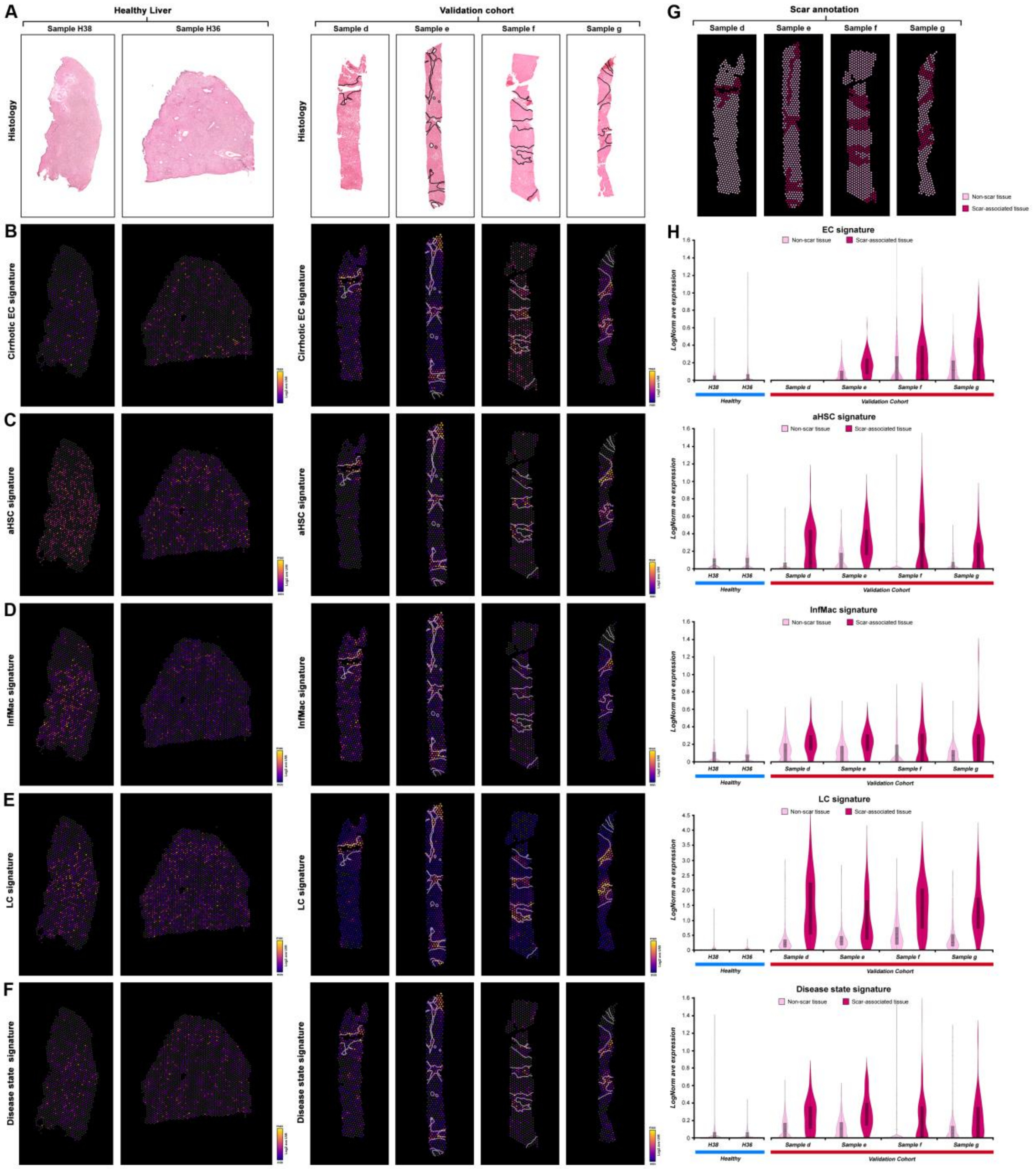

Appendix Figure S10. Quantification of spatial expression signatures in healthy and disease liver.

(**A**) Histology of healthy and fibrotic validation samples. (**B-F**) Comparison of spatial expression of cell type specific gene signatures in healthy and fibrotic samples (log2 average UMIs). (**G**) Spatial annotation of scar-associated (fuchsia) and non-scar tissue (pink). (**H**) Violin plots show expression (log norm average UMIs) of cell type signatures across healthy and fibrotic validation samples.

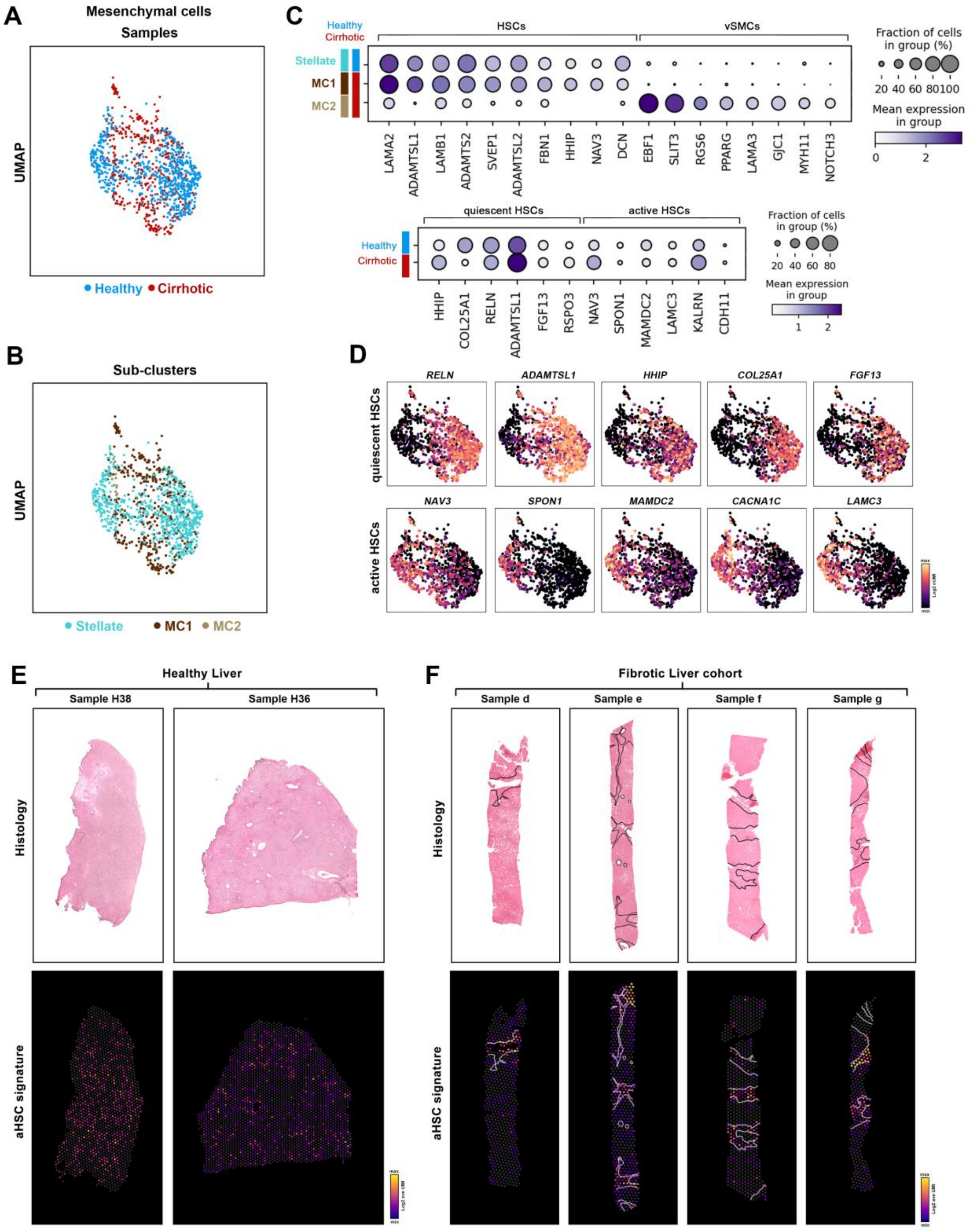

Appendix Figure S11. Mesenchymal cell subpopulations in healthy and cirrhotic liver.

(**A**,**B**) UMAP plots of (**A**) mesenchymal cells (MCs) across healthy and cirrhotic samples and (**B**) nuclei coloured by sub-cluster metadata, spots represent individual nuclei. (**C**) Dot plots comparing mean expression of genes associated with hepatic stellate cells (HSCs), vascular smooth muscle cells (vSMCs) and HSC cell state (active/quiescent) across healthy and cirrhotic sub-populations. (**D**) Expression UMAPs of markers of quiescent/aHSCs (log2 UMIs). Spatial mapping of aHSC signature (log2 average UMIs of *NAV3*, *MAMDC2*, *LAMC3*, *KALRN*, *CDH11*) in (**E**) healthy and (**F**) fibrotic validation samples.

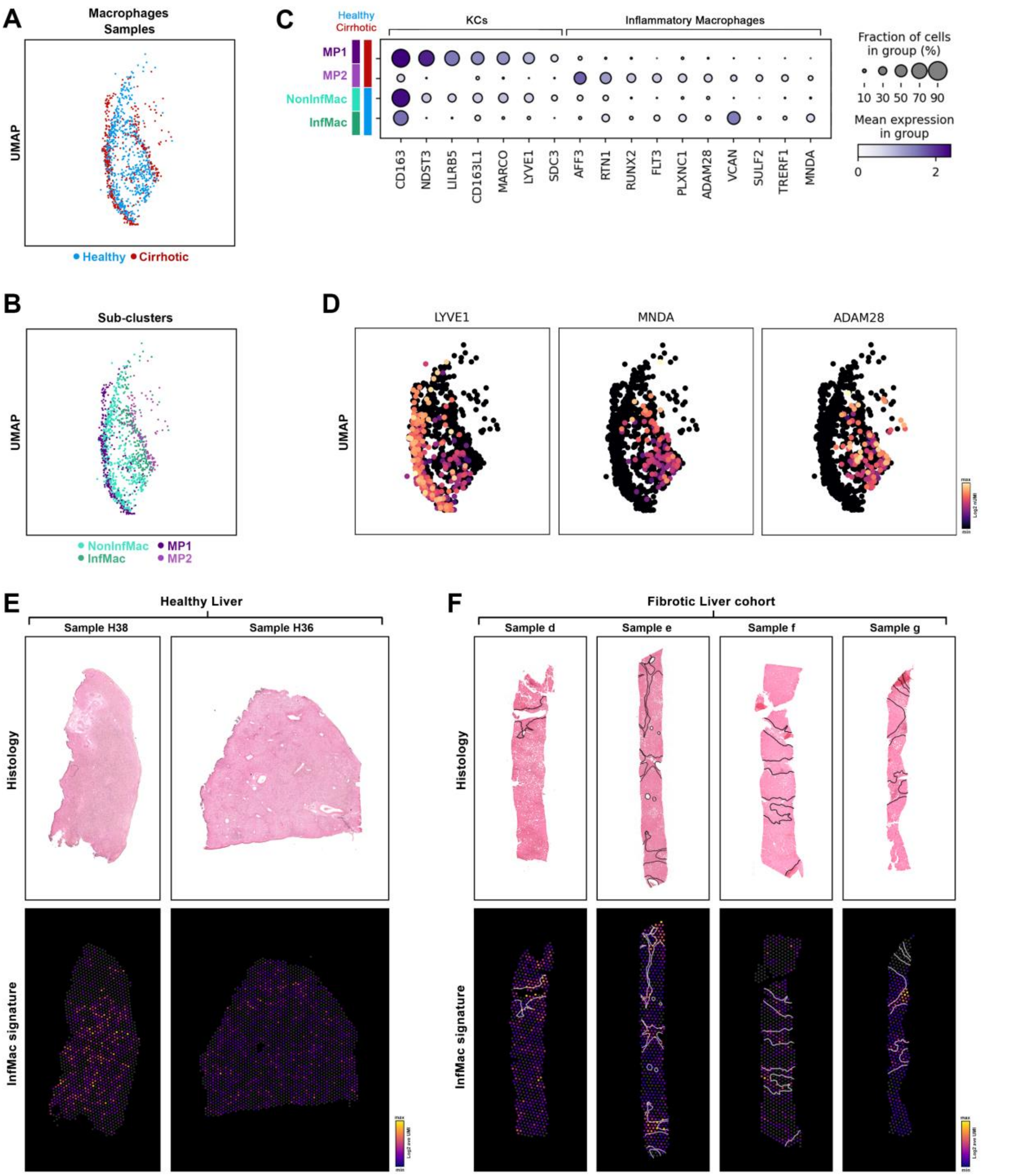

Appendix Figure S12. Macrophage cell subpopulations in healthy and cirrhotic liver.

(**A**,**B**) UMAP plots of (**A**) macrophages (MPs) across healthy and cirrhotic samples and (**B**) nuclei coloured by sub-cluster metadata, spots represent individual nuclei. (**C**) Dot plot comparing mean expression of genes associated with resident macrophages (Kupffer Cells; KCs) and inflammatory macrophages (IMs) across healthy and cirrhotic sub-populations. (**D**) Expression UMAPs *LYVE1*, *MNDA* and *ADAM28*. Spatial mapping of IM signature (log2 average UMIs of *AFF3*, *RTN1*, *FLT3*, *PLXNC1*, *ADAM28*, *VCAN*, *SULF2*, *TRERF1*, *MNDA*) in (**E**) healthy and (**F**) fibrotic validation samples.

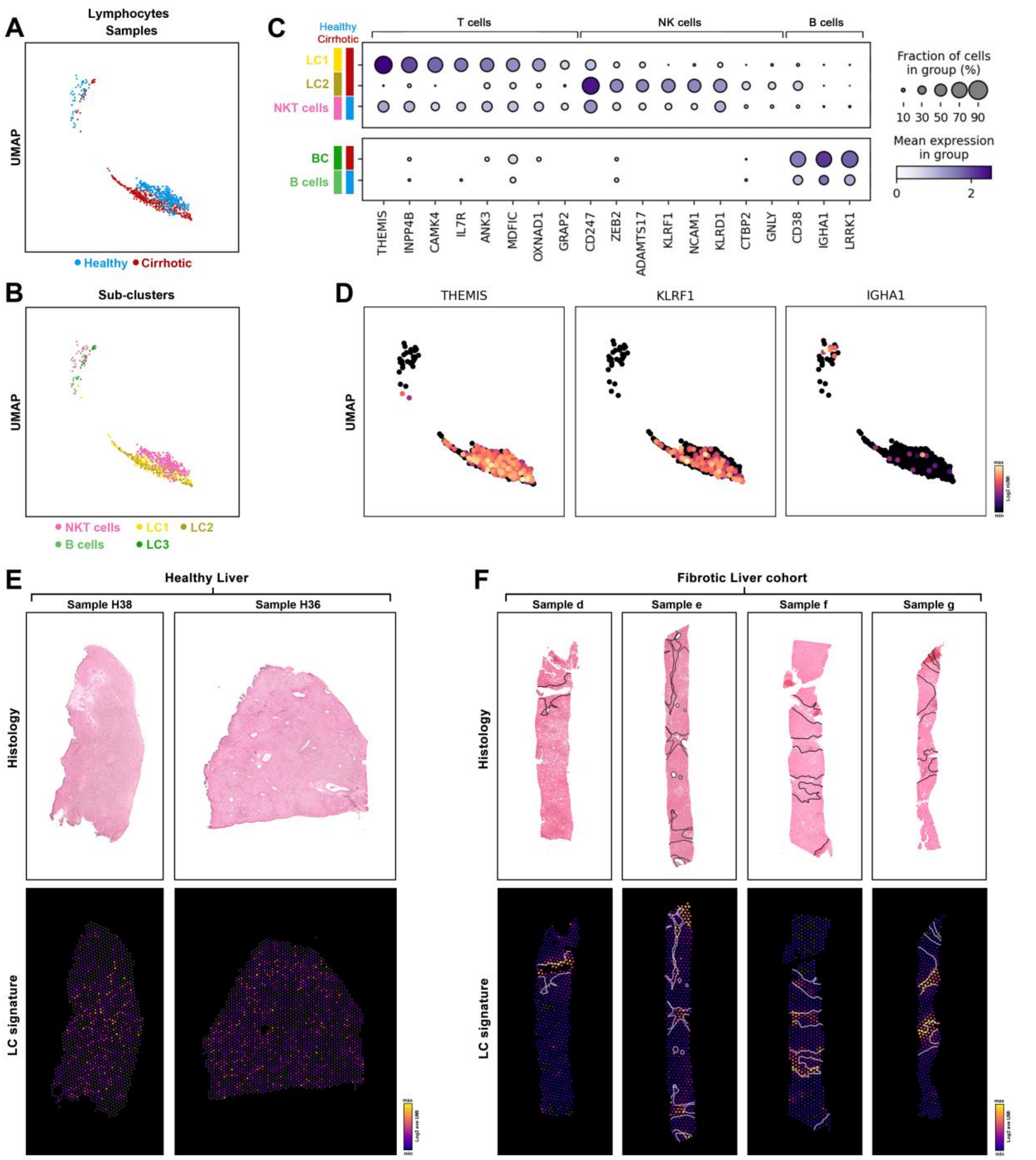

Appendix Figure S13. Lymphocyte cell subpopulations in healthy and cirrhotic liver.

(**A**,**B**) UMAP plots of (**A**) T lymphocytes (LCs) and B lymphocytes (BCs) across healthy and cirrhotic samples, with (**B**) nuclei coloured by sub-cluster metadata, spots represent individual nuclei. (**C**) Dot plot comparing mean expression of genes associated with T cells, natural killer cells (NKs) and BCs across healthy and cirrhotic sub-populations. (**D**) Expression UMAPs of *THEMIS*, *KLRF1* and *IGHA1* (log2 UMIs). Spatial mapping of LC signature (log2 average UMIs of *THEMIS, INPP4B, CAMK4, IL7R, ANK3, MDFIC, OXNAD1, GRAP2, CD247, ZEB2, ADAMTS17, KLRF1, NCAM1, KLRD1, GNLY, CD38, IGHA1, LRRK1*) in (**E**) healthy and (**F**) fibrotic validation samples.

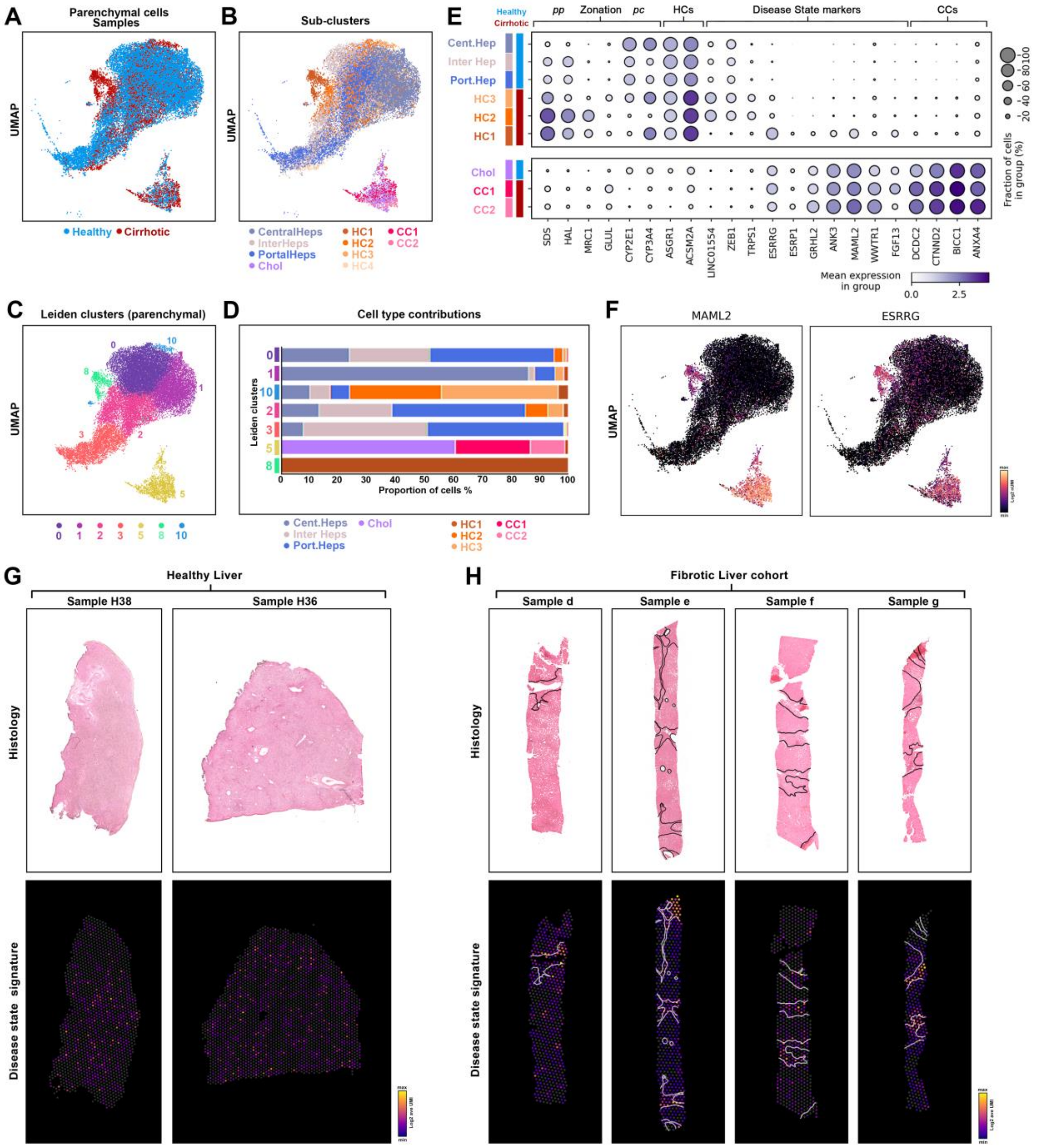

Appendix Figure S14. Parenchymal cell subpopulations in healthy and cirrhotic liver.

(**A**,**B**) UMAP plots of (**A**) hepatocytes (HCs) and cholangiocytes (CCs) across healthy and cirrhotic samples, with (**B**) nuclei coloured by sample sub-cluster and (**C**) Leiden cluster metadata, spots represent individual nuclei. (**D**) Proportion of healthy and cirrhotic cells (%) underlying Leiden clusters, showing the outlier 'cluster 8' uniquely contains cirrhotic HC1 cells. (**E**) Dot plot comparing mean expression of genes associated with hepatocyte zonation, HCs, CCs and the cirrhotic disease state across healthy and cirrhotic sub-populations. (**F**) Expression UMAPs of *MAML2* and *ESRRG* (log2 UMI), spots represent individual nuclei. Spatial mapping of disease state signature (log2 average UMIs of *ANK3*, *GRHL2*, *ARHGEF38*, *MAML2*, *ESRP1*, *ESRRG*, *WWTR1*, *FGF13*) in (**G**) healthy and (**H**) fibrotic validation samples.

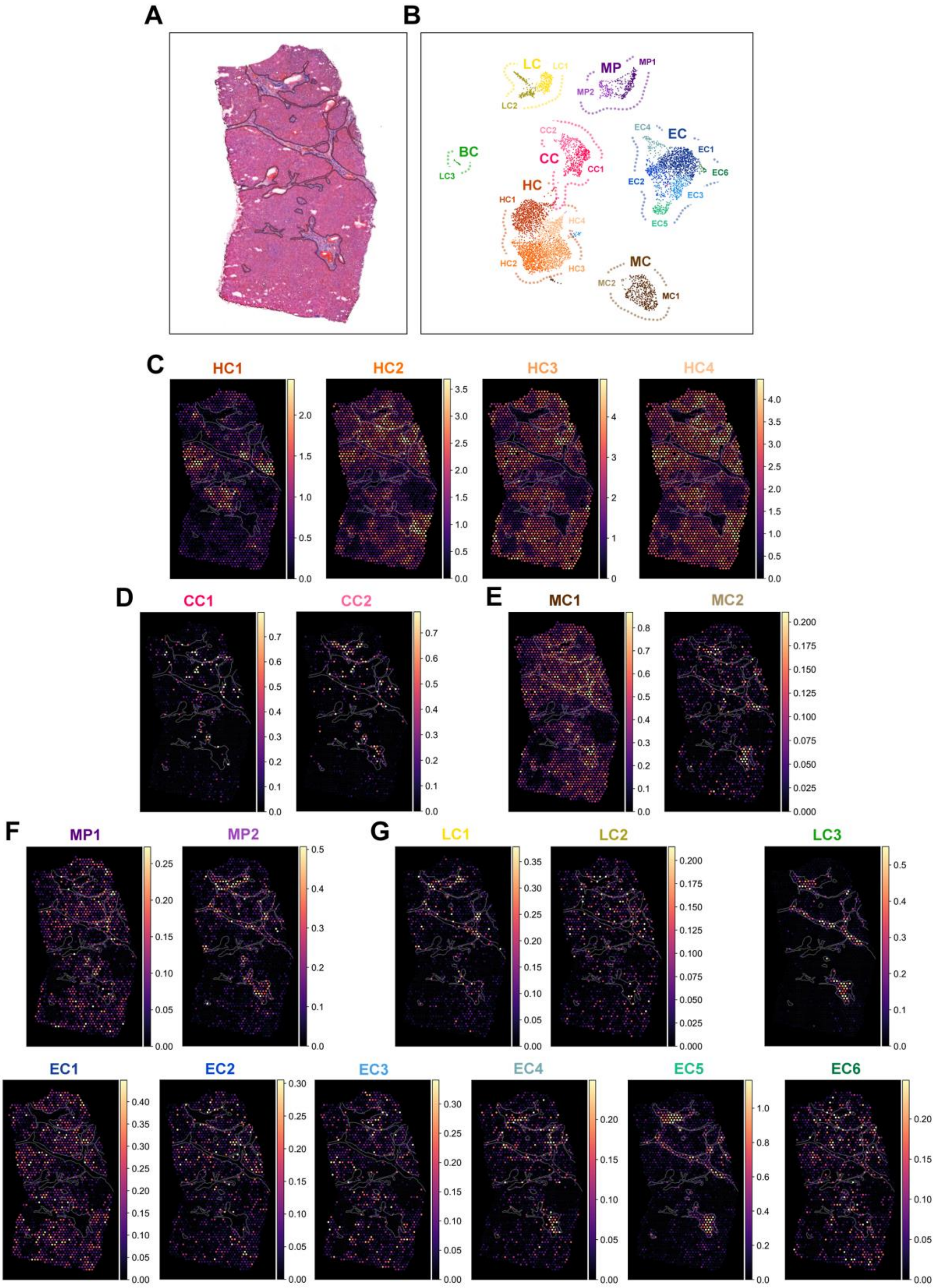

Appendix Figure S15. Deconvolution and spatial mapping of cell type sub-clusters in sample A

(**A**) Brightfield imaging of sample a, annotated to show fibrotic scars. (**B**) snRNA-seq cirrhotic liver cell type sub-clusters were spatially mapped using cell2location, showing regions occupied by hepatocytes (**C**; HC1-4), cholangiocytes (**D**; CC1-2; **D**), mesenchymal cells (**E**; MC1-2), macrophages (**F**; MP1-2), T and B lymphocytes (**G**; LC1-3) and endothelial cells (**H**; EC1-6). Spatial spots show cell abundance (colour intensity) for each sub-cluster.

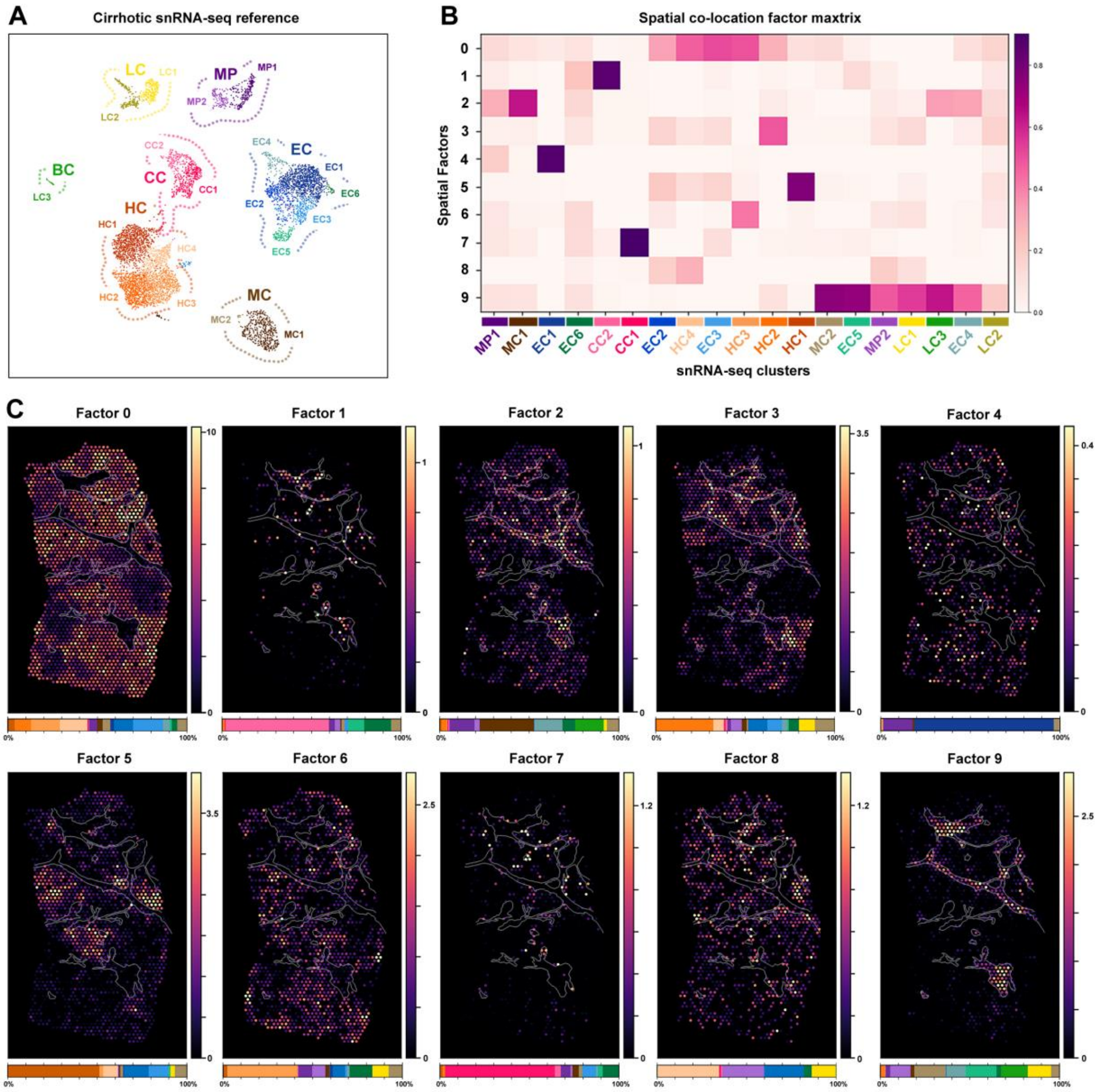

Appendix Figure S16. Co-location analysis of cell type sub-clusters in sample A

(**A**) Reference gene signatures from snRNA-seq subpopulations were used to deconvolute multi-cell ST spots. (**B**) Co-location analysis using estimated non-negative matrix factorisation (NMF) shows 10 factor maps (0-9) and the proportion of cell sub-clusters represented by each spatial factor. (**C**) Spatial mapping of co-location factors (0-9), depicting estimated cell abundance and corresponding bar charts showing percentage of cell subpopulations which represent each factor. Hepatocytes (HC), endothelial cells (EC), cholangiocytes (CC), macrophages (MP), mesenchymal cells (MC), T lymphocytes (LC), and B lymphocytes (BC).

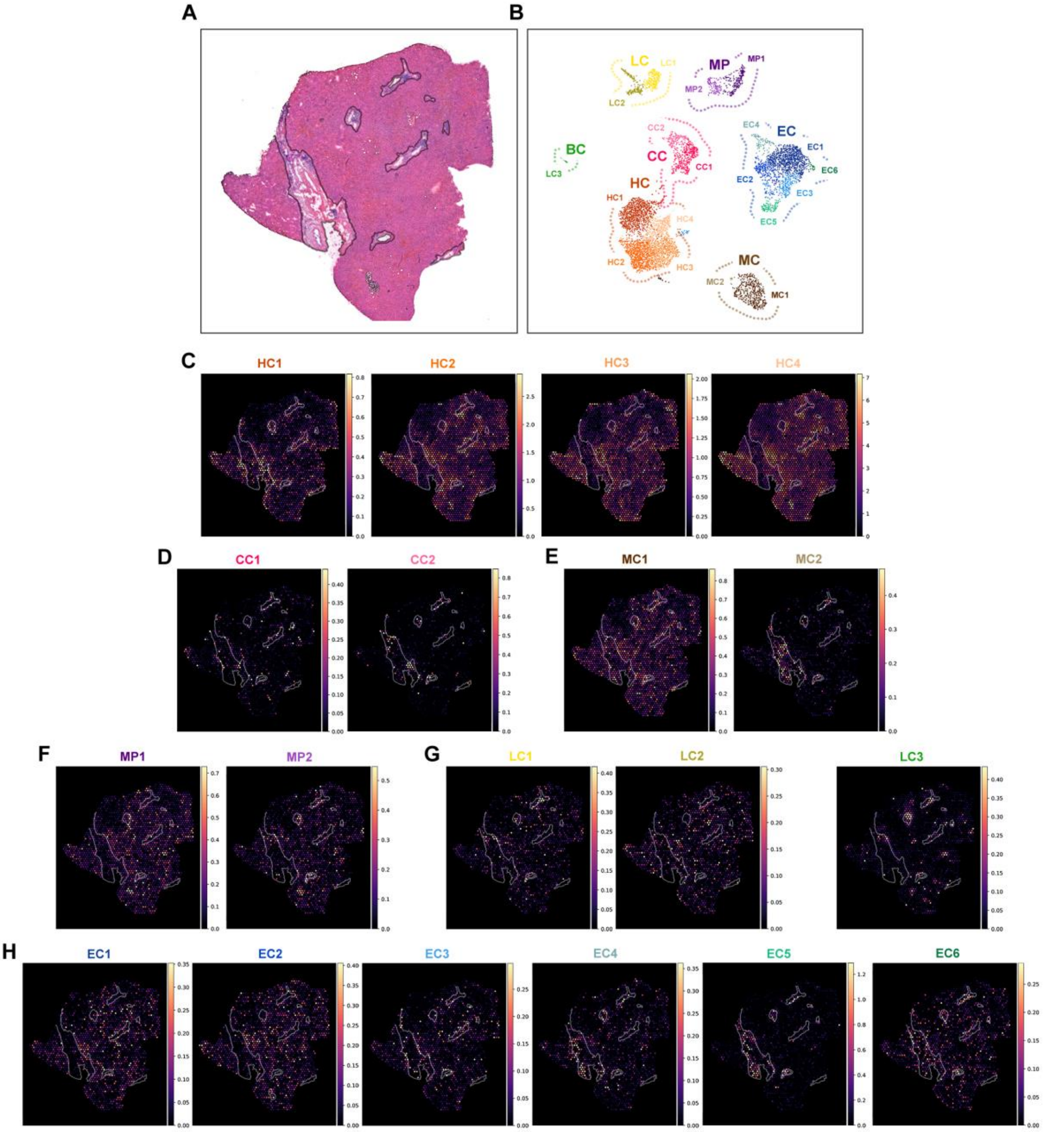

Appendix Figure S17. Deconvolution and spatial mapping of cell type sub-clusters in sample C.

(**A**) Brightfield imaging of sample c, annotated to show fibrotic scars. (**B**) snRNA-seq cirrhotic liver cell type sub-clusters were spatially mapped using cell2location, showing regions occupied by hepatocytes (**C**; HC1-4), cholangiocytes (**D**; CC1-2; **D**), mesenchymal cells (**E**; MC1-2), macrophages (**F**; MP1-2), T and B lymphocytes (**G**; LC1-3) and endothelial cells (**H**; EC1-6). Spatial spots show cell abundance (colour intensity) for each sub-cluster.

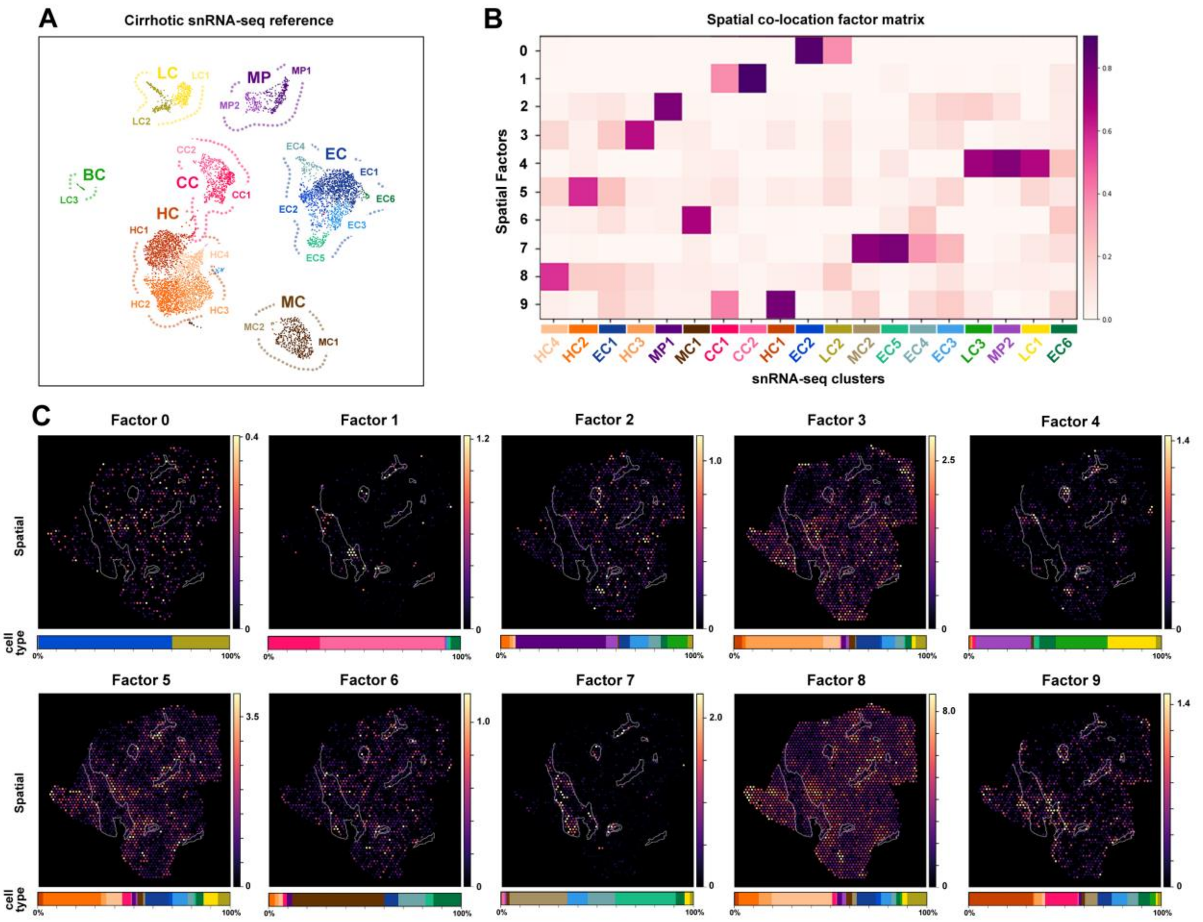

Appendix Figure S18. Co-location analysis of cell type sub-clusters in sample C.

(**A**) Reference gene signatures from snRNA-seq subpopulations were used to deconvolute multi-cell ST spots. (**B**) Co-location analysis using non-negative matrix factorisation (NMF) shows 10 factor maps (0-9) and the proportion of cell sub-clusters represented by each spatial factor. (**C**) Spatial mapping of co-location factors (0-9), depicting estimated cell abundance and corresponding bar charts showing percentage of cell subpopulations which represent each factor Hepatocytes (HC), endothelial cells (EC), cholangiocytes (CC), macrophages (MP), mesenchymal cells (MC), T lymphocytes (LC), B lymphocytes (BC).

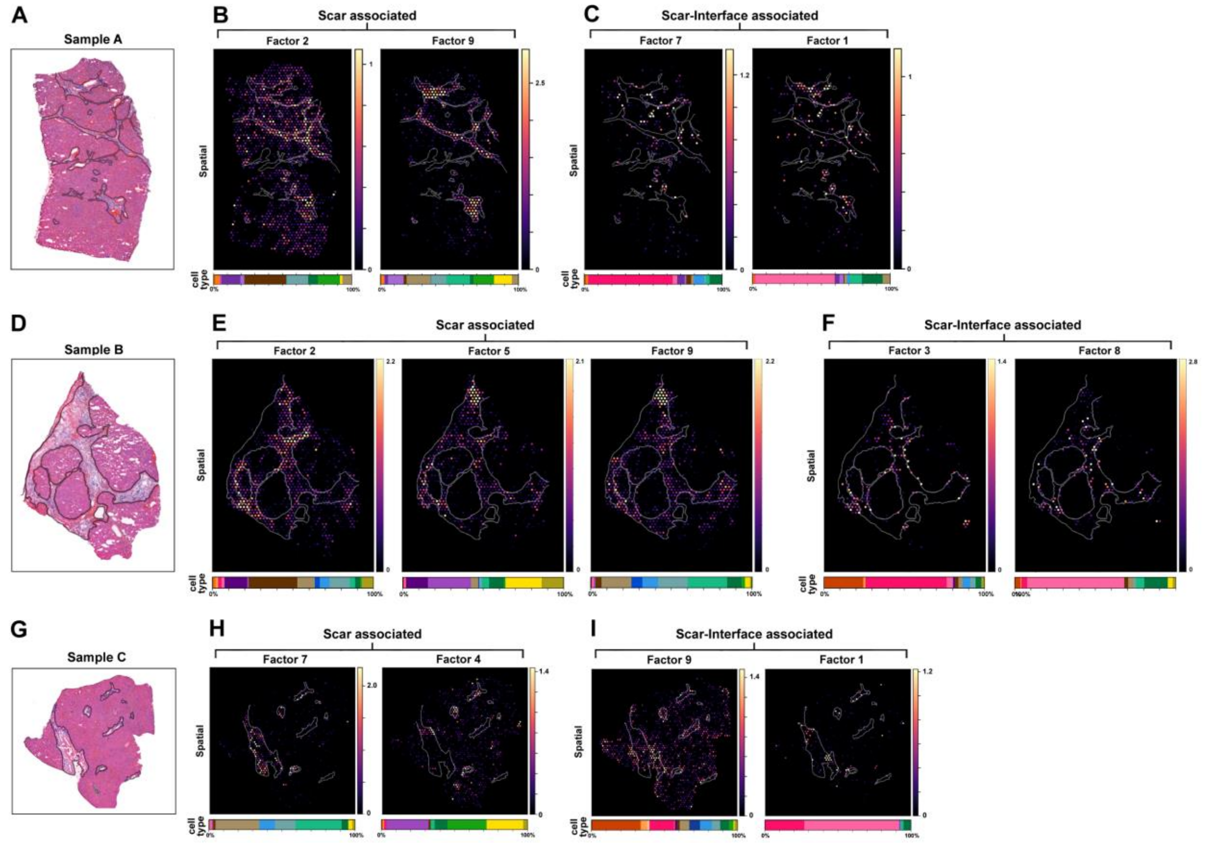

Appendix Figure S19. Scar and scar-interface associated co-location factors across experimental cohort patient samples.

(**A-I**) Histology of spatial samples from patients diagnosed with cirrhosis in experimental cohort (**A, D, G**) with fibrotic scars outlined. Reference snRNA-seq signatures were used to deconvolute spatial spots, per sample, followed by co-location factor analysis, enabling a comparison of scar associated (**B, E, H**) and scar-interface associated factors across samples. Spatial spots show estimated cell abundance (colour intensity) for each co-location factor, which together with bar charts show percentage of cell sub-types for each factor.

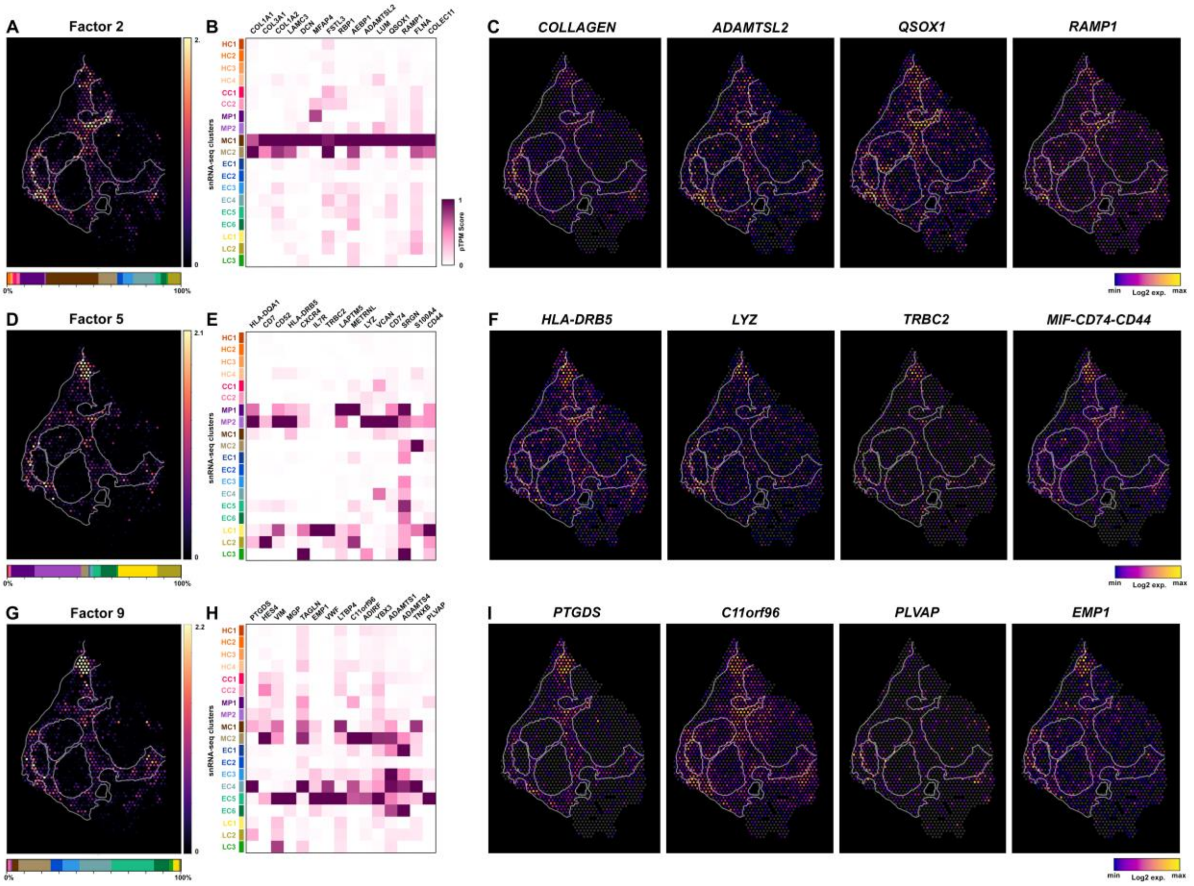

Appendix Figure S20. Non-parenchymal cell sub-populations are co-localised within the fibrotic niche.

(**A-I**) Spatial deconvolution of non-parenchymal cell types show several sub-populations are co-localised (Factors 2, 5 and 9) within the fibrotic scar (**A**,**D**,**G**). Selected DEGs of Factors 2, 5 and 9 (**B**,**E**,**H**) are correlated with their snRNA-seq expression score and reveal the spatial expression (log2 UMIs) of scar-associated targets (**C**,**F**,**I**). Hepatocytes (HC), endothelial cells (EC), cholangiocytes (CC), macrophages (MP), mesenchymal cells (MC), T and B lymphocytes (LC). Spatial factors show estimated cell abundance (spot colour intensity) for each co-location factor, which together with bar charts show percentage of cell sub-types for each factor (**A**,**D**,**G**).

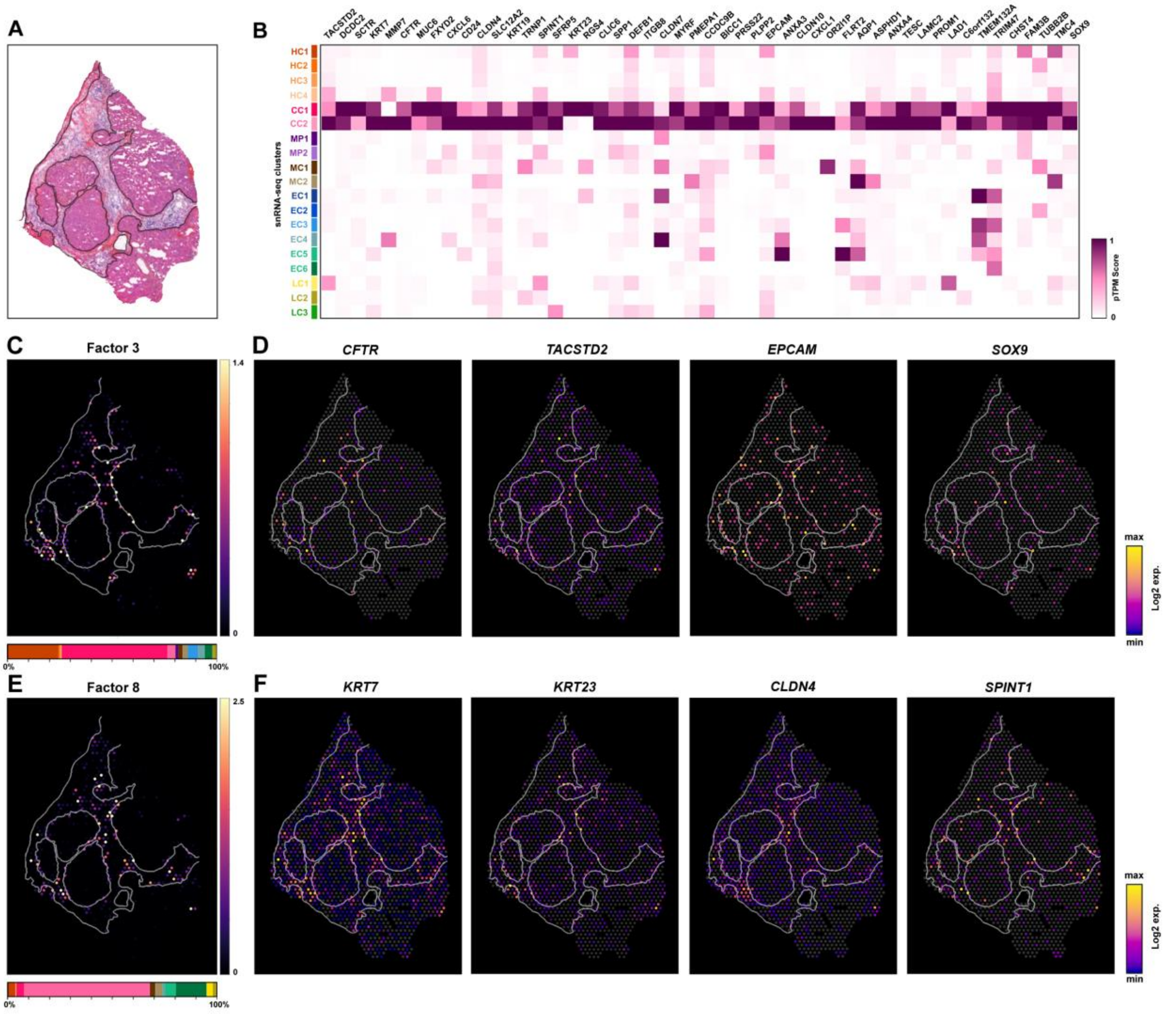

Appendix Figure S21. Disease-associated hybrid parenchymal cells are co-localised to the scar-interface.

(**A-F**) Spatial deconvolution of parenchymal sub-populations reveal HC1/CC1 cells are co-localised at the scar-interface (**C**; Factor 3) whereas CC2 sub-populations are preferentially located within and at the scar edge (**E**; Factor 8). Selected DEGs underlying Factors 3 and 8 (**B**) reveal spatial expression (log2 UMIs) of liver progenitor cell (LPC) and cholangiocyte markers enriched at scar-interface (**D**,**F**). Hepatocytes (HC), endothelial cells (EC), cholangiocytes (CC), macrophages (MP), mesenchymal cells (MC), T and B lymphocytes (LC). Spatial factors show estimated cell abundance (spot colour intensity) for each co-location factor, which together with bar charts show percentage of cell sub-types for each factor (**A**,**C**,**E**).

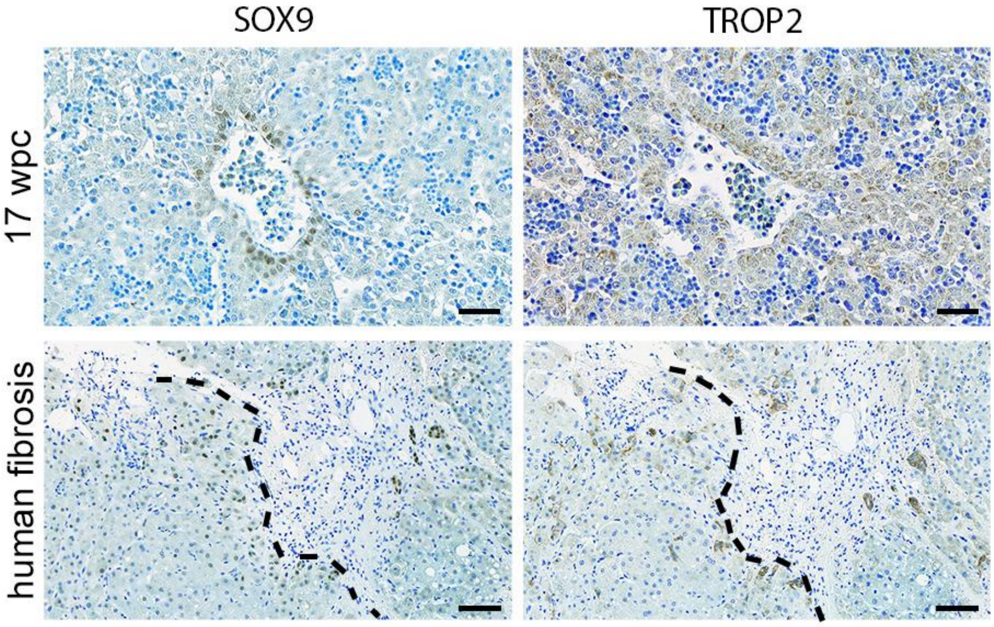

Appendix Figure S22. SOX9 and TROP2 localisation in human development and adult fibrotic liver.

Immunohistochemistry for SOX9 and TROP2 (brown) in human development at 17 wpc (top panel) and liver fibrosis (bottom panel). Dotted line demarcates fibrotic scar edge. Counterstain is toluidine blue and Size bar = 50µm.

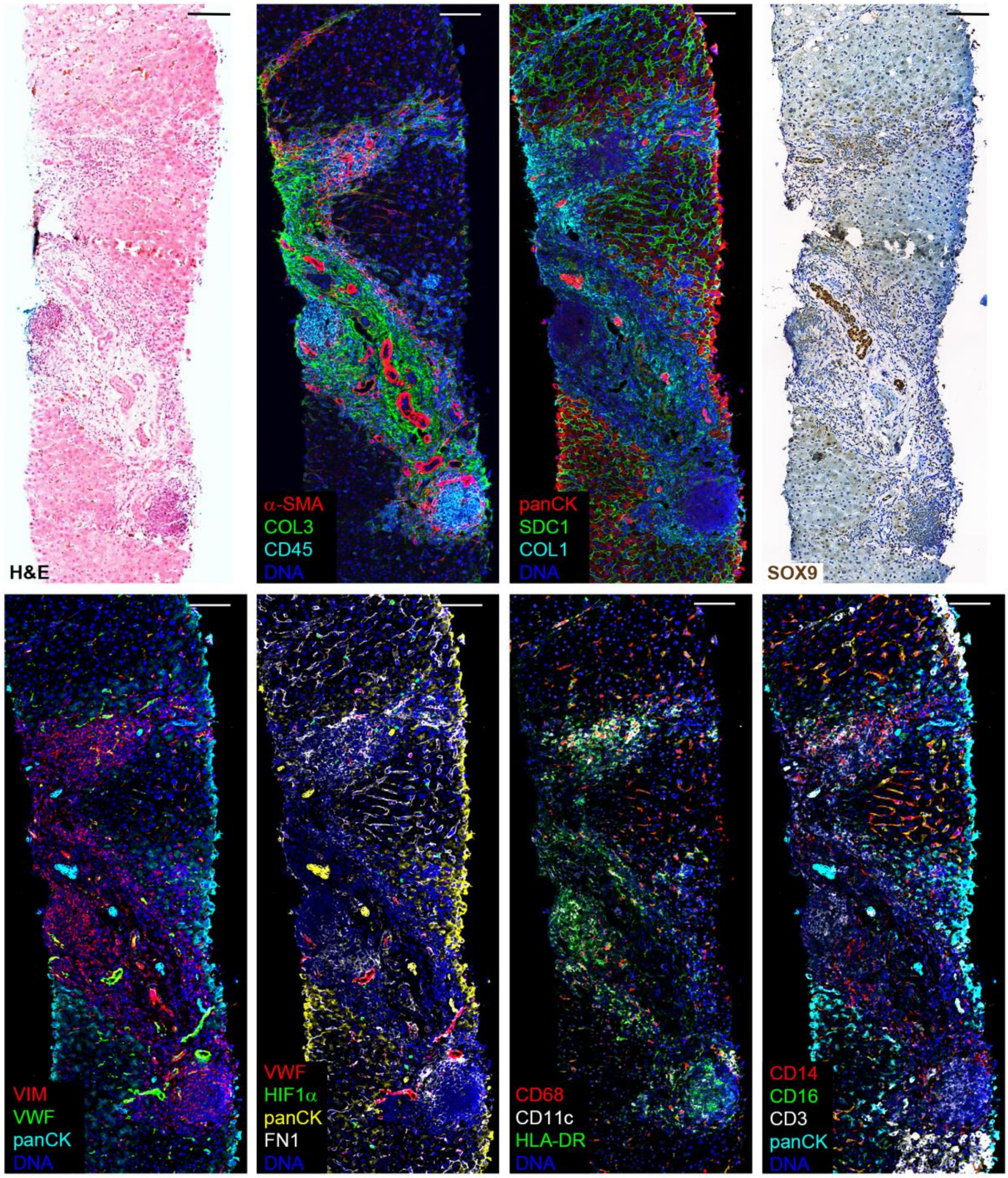

Appendix Figure S23. Characterisation of spatial features with imaging mass cytometry (IMC).

Serial sections of whole biopsy (linked to higher magnification image in Figure 7; n=1) showing imaging mass cytometry (IMC) and bright field images for SOX9 immunohistochemistry (IHC) and H&E staining as indicated. Size bar = 200µm.

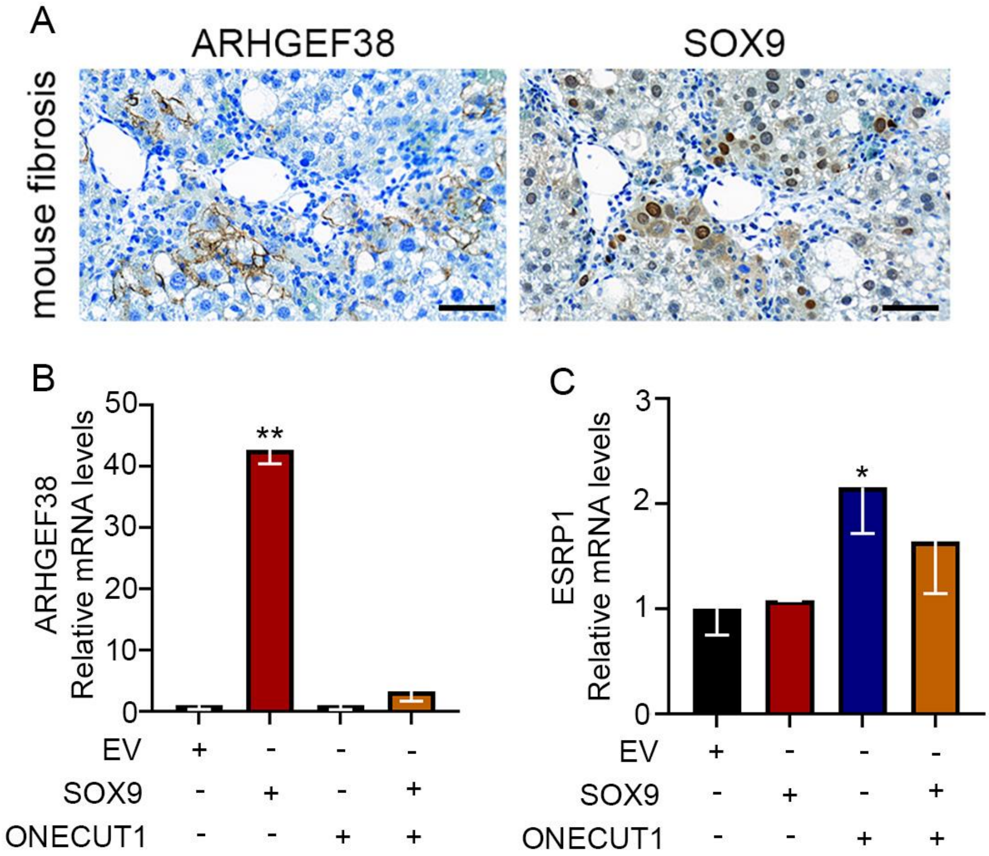

Appendix Figure S24. Gene expression driving disease associated hepatocytes.

(**A**) Serial sections of mouse liver fibrosis induced by 16 week CCl_4 intraperitoneal injection. Immunohistochemistry for ARHGEF38 and SOX9 (brown) counterstained with toluidine blue, Size bar = 25µm. (**B-C**) Overexpression of SOX9 and ONECUT1 in HepG2 cells and relative mRNA expression of ARHGEF38 (B) or ESRP1 (C). Data in bar charts shows mean ± s.e.m. (n=5). One way ANOVA with Dunnett’s multiple comparisons test was used for statistical analysis. *P*-values are **P*=0.0159, ***P*=0.0022.

Metal channel	Antigen	Clone/catalog #	Supplier
**89**	αSMA (1:100)	MCA5781GA	Bio-Rad
**113**	CD68 (1:100)	MA5-13324	Thermo
**139**	PanCK (1:100)	914204	BioLegend
**143**	Vimentin (VIM) (1:100)	3143029D	Fluidigm
**144**	CD14 (1:100)	56082BF	Cell Signalling
**146**	CD16 (1:100)	ab243925	Abcam
**152**	CD45 (1:100)	14-9457-82	eBioscience
**154**	CD11c (1:100)	ab216655	Abcam
**155**	HIF1α (1:100)	ab210073	Abcam
**159**	Von Willebrand Factor (VWF) (1:100)	A0082	Dako
**164**	Syndecan (SD1) (1:100)	ab226108	Abcam
**166**	Collagen III (COL3) (1:100)	ab7778	Abcam
**167**	Fibronectin (FN1) (1:100)	NBP2-34526	Novus
**169**	Collagen I (COL1) (1:100)	3169023D	Fluidigm
**170**	CD3 (1:100)	3170019D	Fluidigm
**174**	HLA-DR (1:100)	ab176408	Abcam

Appendix Table S1.

Antibody panel for IMC.
