# Supplementary figures and images for: Spatial gene regulatory networks driving cell state transitions during human liver disease
Source: EMBO Mol Med. 2025 Apr 25;17(6):1452–74. doi: 10.1038/s44321-025-00230-6 (PMC12162837; doi:10.1038/s44321-025-00230-6)

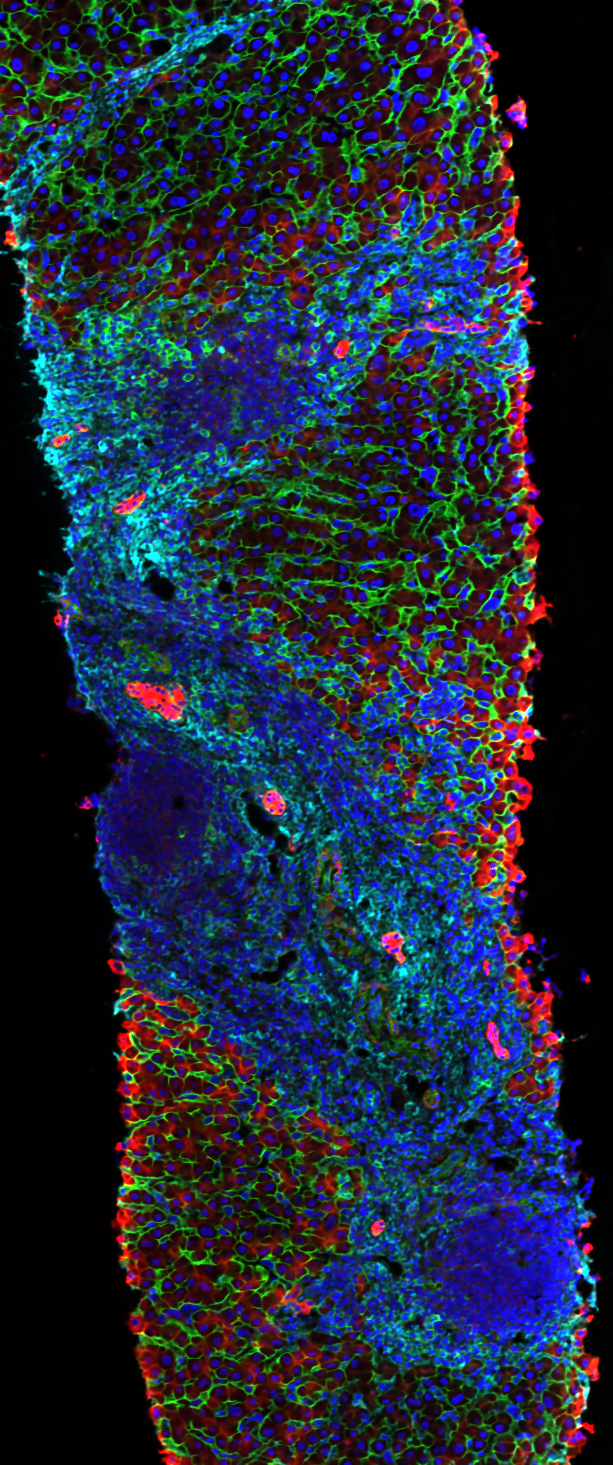

Supplement: Supplementary file 12 — Source data Fig. 7 [file 44321_2025_230_MOESM12_ESM.zip › Source files/COL1 cyan panCK red SDC1 green DNA blue.tif (RGB).tif]

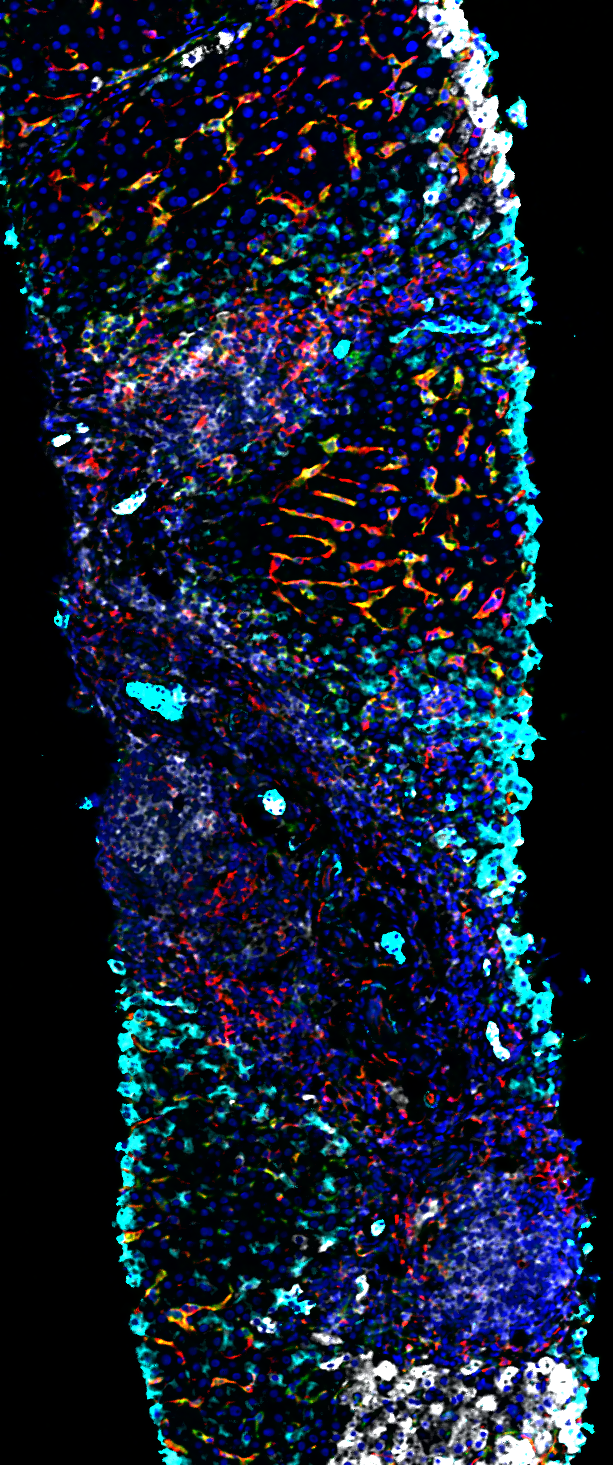

Supplement: Supplementary file 12 — Source data Fig. 7 [file 44321_2025_230_MOESM12_ESM.zip › Source files/DNA CD14red CD16green CD3white panCKcyan.tif (RGB).tif]

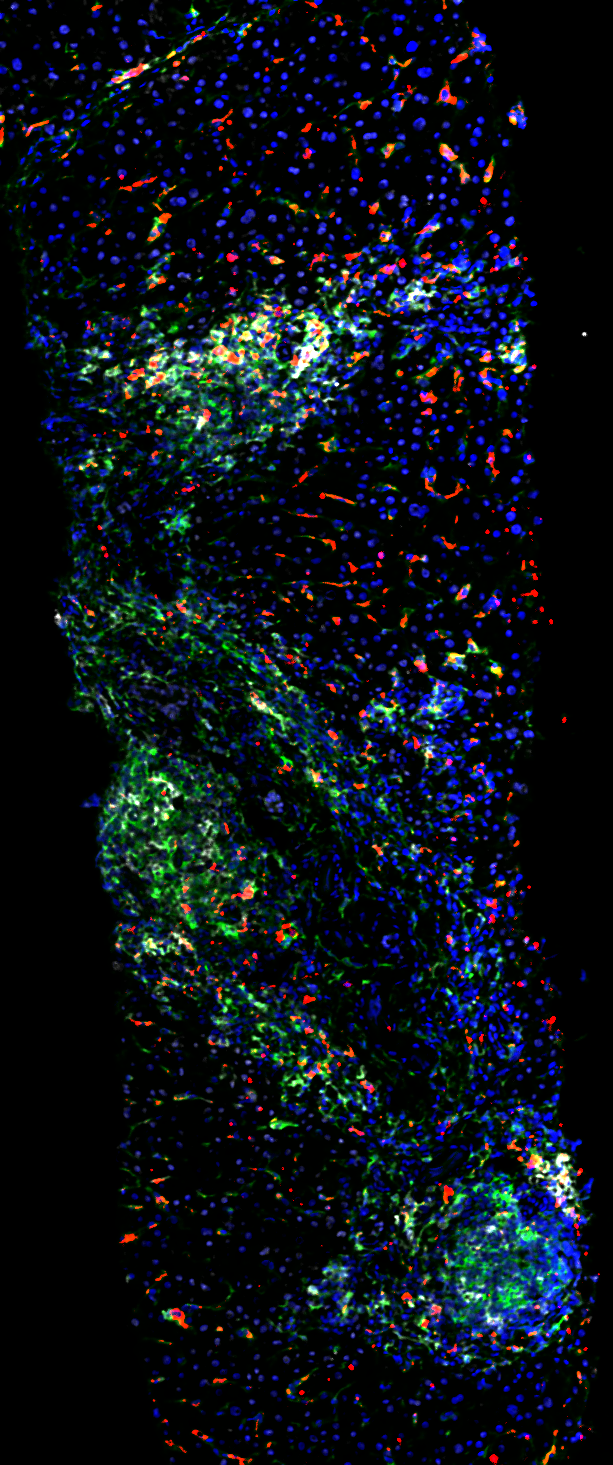

Supplement: Supplementary file 12 — Source data Fig. 7 [file 44321_2025_230_MOESM12_ESM.zip › Source files/DNAblue CD68red CD11cwhite HLADRgreen.tif (RGB).tif]

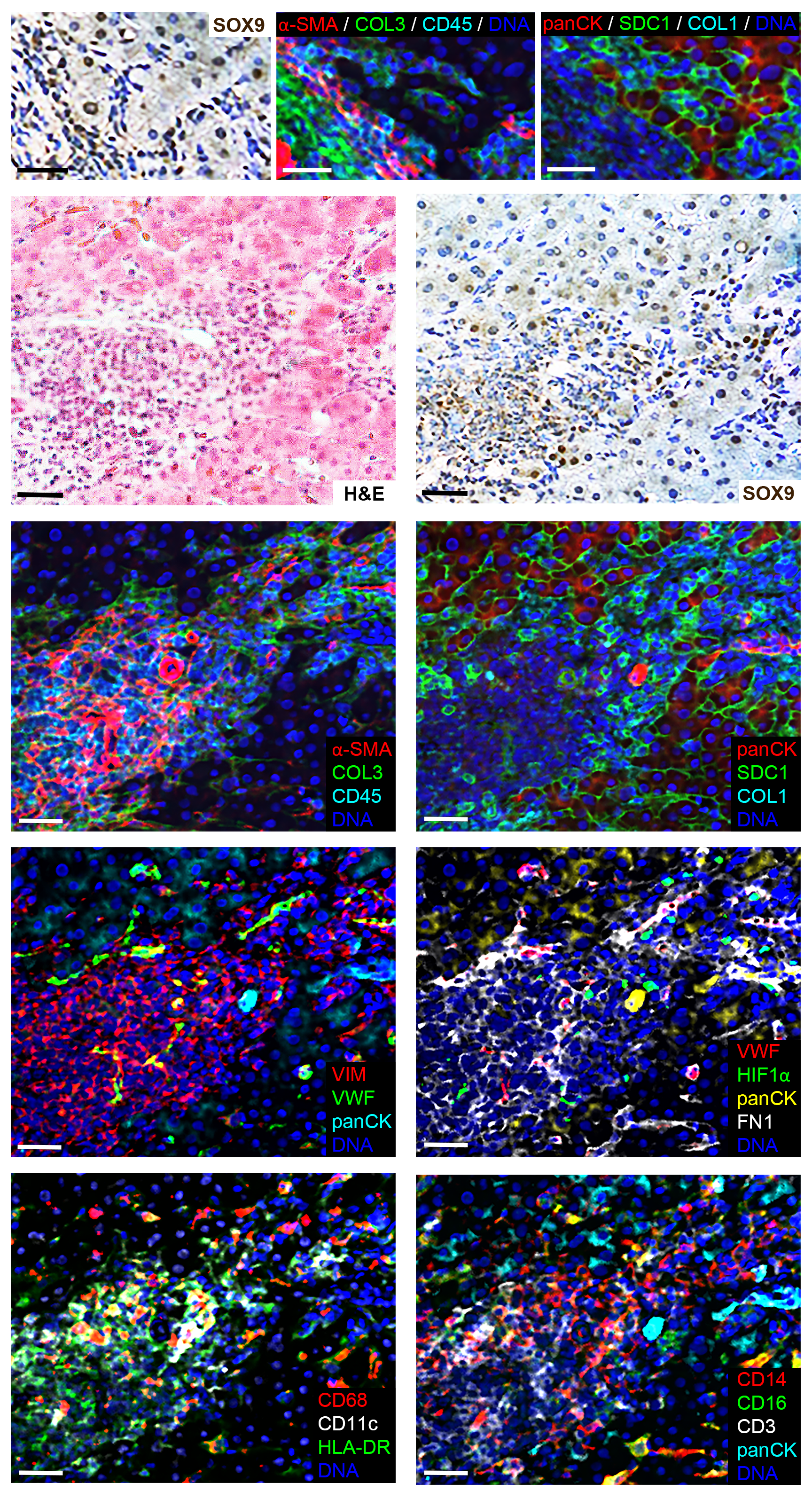

Supplement: Supplementary file 12 — Source data Fig. 7 [file 44321_2025_230_MOESM12_ESM.zip › Source files/Figure 7 source file.tif]

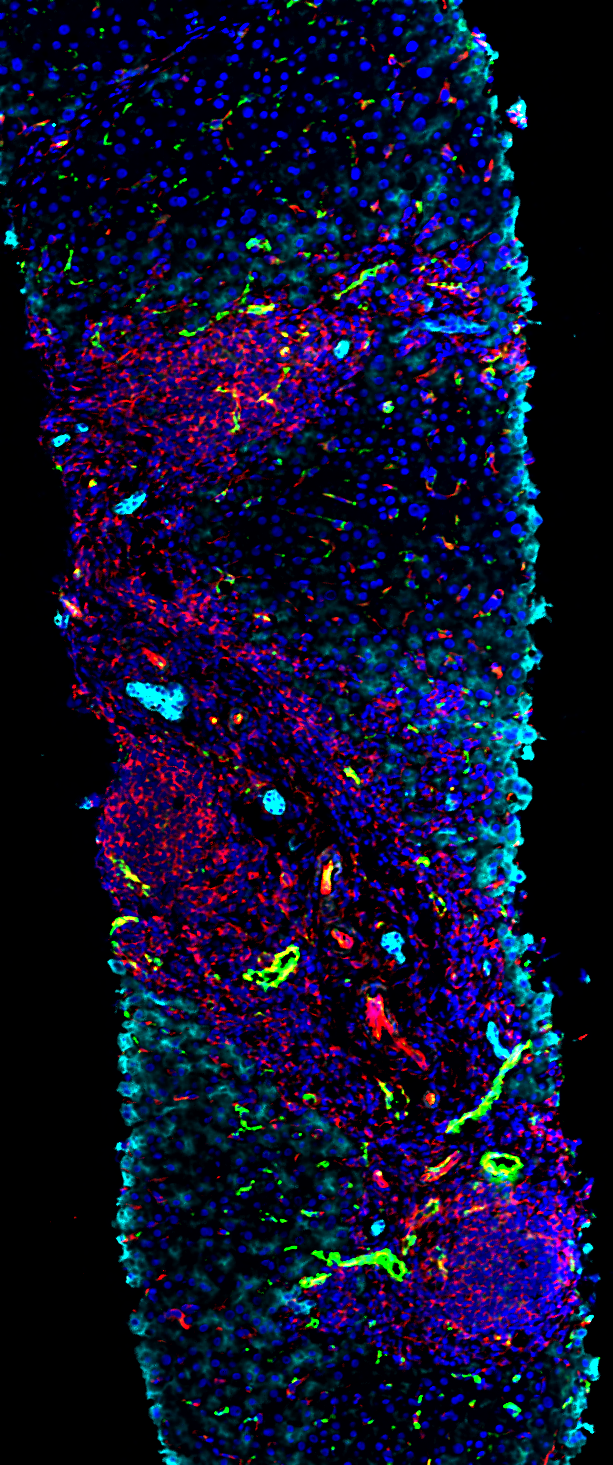

Supplement: Supplementary file 12 — Source data Fig. 7 [file 44321_2025_230_MOESM12_ESM.zip › Source files/PanCK cyan Vim red VWF green DNA blue.tif (RGB).tif]

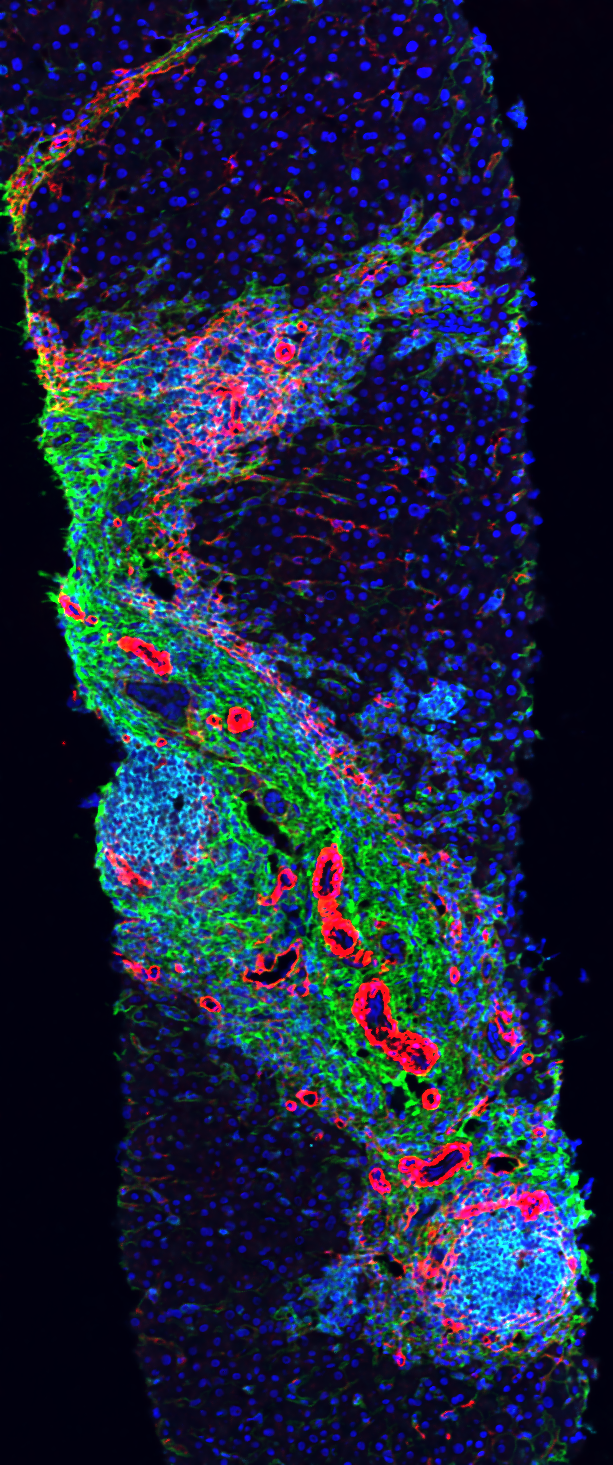

Supplement: Supplementary file 12 — Source data Fig. 7 [file 44321_2025_230_MOESM12_ESM.zip › Source files/Sma red COL3 green CD45 blue and DNA.tif (RGB).tif]

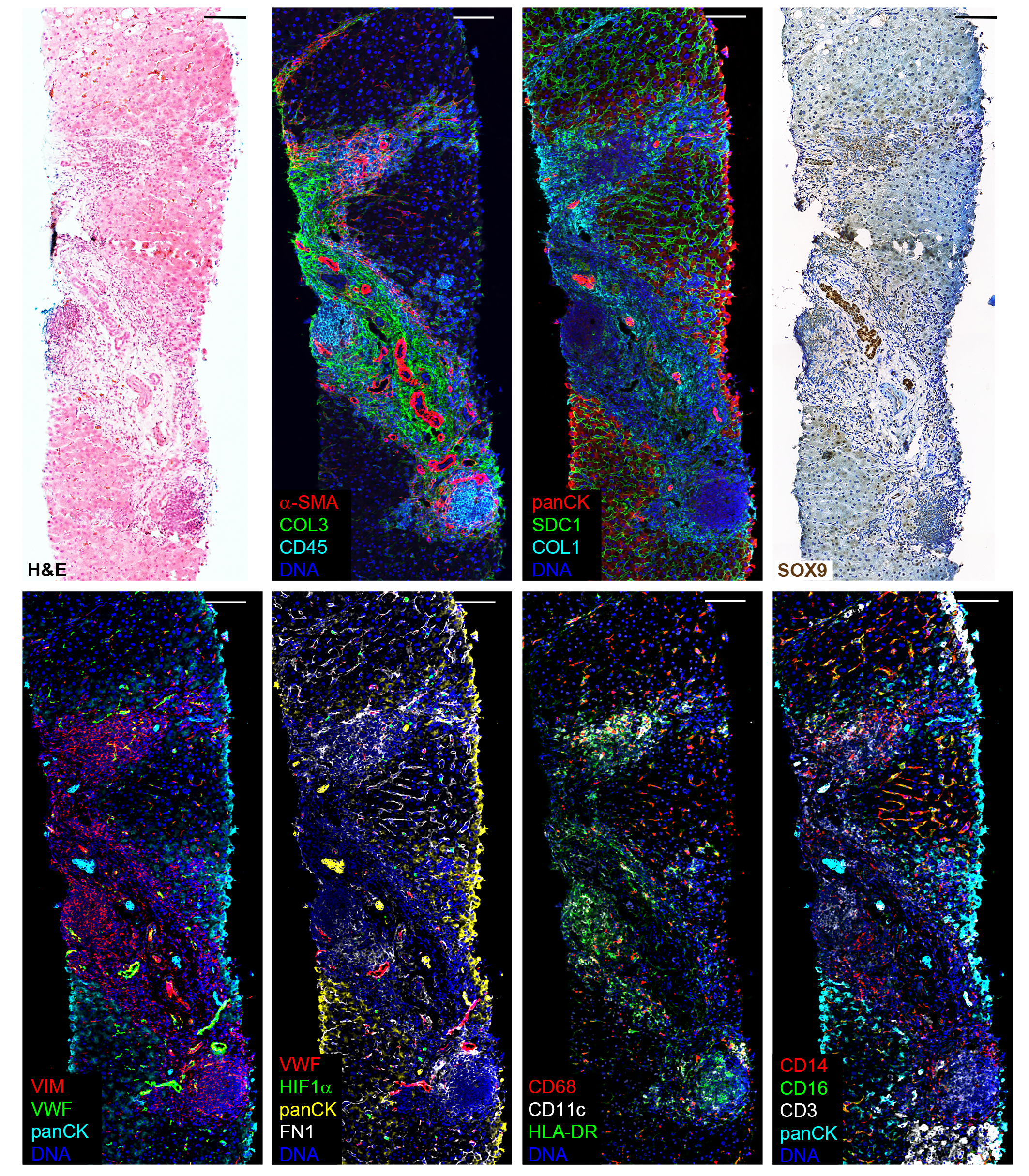

Supplement: Supplementary file 12 — Source data Fig. 7 [file 44321_2025_230_MOESM12_ESM.zip › Source files/Supplementary Figure 23 source file.tif]

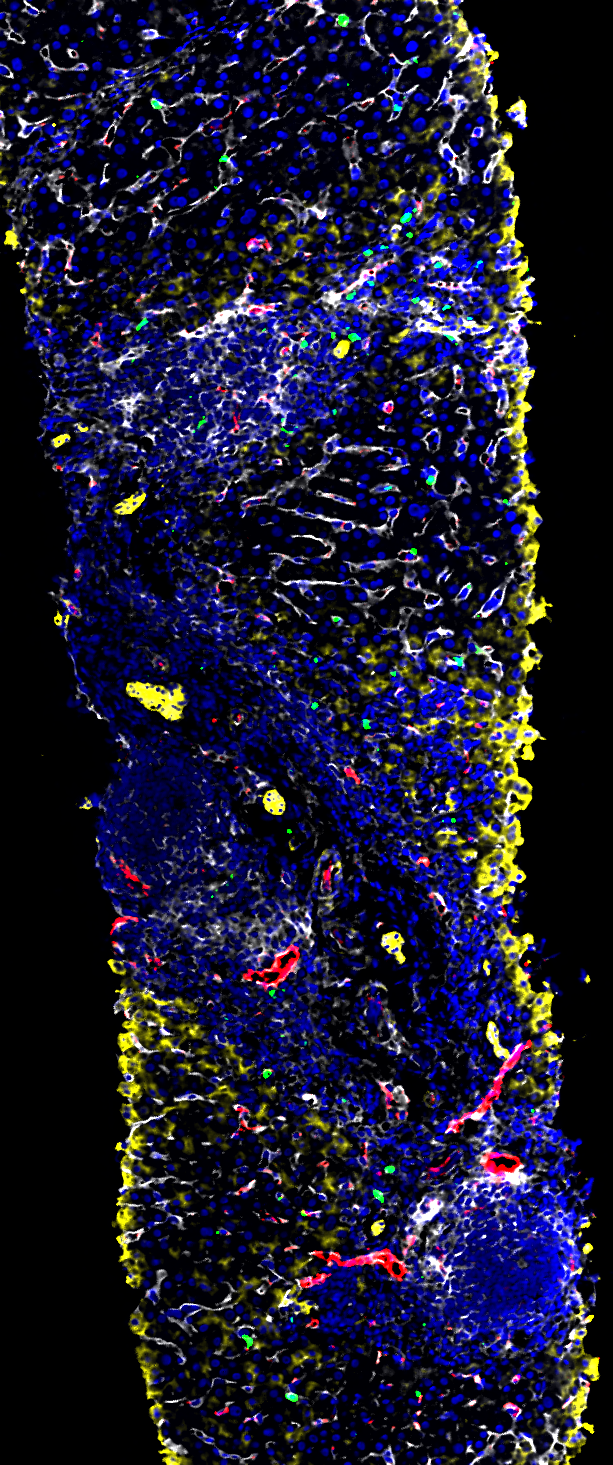

Supplement: Supplementary file 12 — Source data Fig. 7 [file 44321_2025_230_MOESM12_ESM.zip › Source files/VWF red panCK yellow FN1 white HIF1 green DNA blue.tif (RGB).tif]
